# Supplementary material for: Prime editing corrects the dilated cardiomyopathy causing RBM20-P633L-mutation in human cardiomyocytes
Source: Mol Ther Nucleic Acids. 2025 Oct 6;36(4):102734. doi: 10.1016/j.omtn.2025.102734 (PMC12590231; doi:10.1016/j.omtn.2025.102734)
Supplement: Document S1. Figures S1–S5 and Tables S1–S3 [file mmc1.pdf]

OMTN, Volume *36*

Supplemental information

Prime editing corrects the dilated cardiomyopathy causing RBM20-P633L-mutation in human cardiomyocytes

Alexandra Roman, Anja Zimmer, Michael Gotthardt, Lars M. Steinmetz, Ralf Kühn, and Tu Dang

Supplemental Tables

Table S1 Sequences of pegRNAs

pegRNA#	Spacer		Extension		
	#	sequence (5’>3’)	RT [bp]	PBS [bp]	sequence (5’>3’)
RBM20-01	1 (PAM disrupting)	CTCACAGATA TGGCCCAGAA	14	14	GACCGCGGCCTTTCTGGCCATATCT GT
RBM20-02			26	13	ACCGGACTACGAGACcGCgGCCTTTCT GGGCCATATCTG
RBM20-03			18	10	ACGAGACCGCGGtCTTTC GGGCCATAT
RBM20-04			21	10	ACTACGAGACCGCGGTCTTTC TGGGCCATAT
RBM20-05			24	10	CGGACTACGAGACCGCGGTCTTTCTG GGCCATAT
RBM20-06			18	13	ACGAGACCGCGGTCTTTCTGGGCCAT ATCTG
RBM20-07			21	13	ACTACGAGACCGCGGTCTTTCTGGGC CATATCTG
RBM20-08			24	13	CGGACTACGAGACCGCGGTCTTTCTG GGCCATATCTG
RBM20-09			18	15	ACGAGACCGCGGTCTTTCTGGGCCAT ATCTGTG
RBM20-10			21	15	ACTACGAGACCGCGGTCTTTCTGGGC CATATCTGTG
RBM20-11			24	15	CGGACTACGAGACCGCGGTCTTTCTG GGCCATATCTGTG
RBM20-12	2	GGGGAGAGT GACCGGCTCA C	24	10	AGGCCGCGGTCTCGTAGTCCGGTGAG CCGGTCAC
RBM20-13			22	12	GCCGCGGTCTCGTAGTCCGGTGAGCC GGTCACTC
RBM20-14	3	CTTGGCTCCC TCACAGATA	38	15	TCACCGGACTACGAGACcGCgGCCTTT CTGGGCCATATCTGTGAGGGAGCCAA

RBM20-15			38	14	TCACCGGACTACGAGACcGCgGCCTTTCTGGGCCATATCTGTGAGGGAGCCA
RBM20-16			38	13	TCACCGGACTACGAGACcGCgGCCTTTCTGGGCCATATCTGTGAGGGAGCC
LMNA-01	1	CAAAGTGCGTGAGGAGTTTA	13	15	TTCAGCTCCTTAAACTCCTCACGCACTT
LMNA-02	2	GGAGCTGAGCAAAGTGCGTG	22	12	TTCAGCTCCTTAAACTCCTCACGCACTTTGCTCA
LMNA-03	3 (PAM disrupting)	GCGTGAGGAGTTTAAGGAGC	12	13	GCGCTTTCAGCTCCTTAAACTCCTC
LMNA-04			15	14	ACCGCGCTTTCAGCTCCTTAAACTCCTCA
LMNA-05			15	13	ACCGCGCTTTCAGCTCCTTAAACTCCTC
LMNA-07			13	10	CGCGCTTTCAGCTCCTTAAACTC
LMNA-08			16	10	CACCGCGCTTTCAGCTCCTTAAACTC
LMNA-09			19	10	ACTCACCGCGCTTTCAGCTCCTTAAACTC
LMNA-10			13	13	CGCGCTTTCAGCTCCTTAAACTCCTC
LMNA-11			16	13	CACCGCGCTTTCAGCTCCTTAAACTCCTC
LMNA-12			19	13	ACTCACCGCGCTTTCAGCTCCTTAAACTCCTC
LMNA-13			13	15	CGCGCTTTCAGCTCCTTAAACTCCTCAC
LMNA-14			16	15	CACCGCGCTTTCAGCTCCTTAAACTCCTCAC
LMNA-15			19	15	ACTCACCGCGCTTTCAGCTCCTTAAACTCCTCAC
LMNA-16			12	15	GCGCTTTcAGCTCCTTAAACTCCTCAC
LMNA-17			12	14	GCGCTTTcAGCTCCTTAAACTCCTCA
LMNA-18			15	15	ACCGCGCTTTcAGCTCCTTAAACTCCTCAC

Venus-01	1	GACGTAGCCT TCGGGCATGG	36	11	ACCACATGAAGcAGCACGACTTCTTCA AGTCCGCCATGCCCGAAGGC

Table S2 Primers

Name	Sequence (5’ > 3’)
IVT_PEmax_Fwd	TCGAGCTCGGTACCTAATACGACtCACTATAAGG
IVT_Rev	(T_{120})CTTCCTACTCAGGCTTTATTCAAAGACCA
epegRNA_Fwd	AATTCAGTCGACTGGATCCG
epegRNA_hRBM20-sg1-18RT-10PBS-Linker-rev	AGGGGGGGGGCTCCTGAATATGGCCCAGAAAG
epegRNA_hRBM20-sg2-24RT-10PBS-Linker-rev	AGGGGGGGGGCTCCTGAGTGACCGGCTCACCG
epegRNA_hRBM20-sg3-38RT-14PBS-Linker-rev	AGGGGGGGGGCTCCTGATGGCTCCCTCACAGA
epegRNA_LMNA_sg3-12RT-13PBS-Linker-Rev	AGGGGGGGGGCTCCTGAGAGGAGTTTAAGGAG
epegRNA_LMNA_sg3-19RT-10PBS-Linker-Rev	AGGGGGGGGGCTCCTGAGAGTTTAAGGAGCTG
epegRNA_LMNA_sg3-12RT-15PBS-Linker-Rev	AGGGGGGGGGCTCCTGAGTGAGGAGTTTAAGG
epegRNA_LMNA_sg3-12RT-14PBS-Linker-Rev	AGGGGGGGGGCTCCTGATGAGGAGTTTAAGGA
U6+AscI-Fw	CATGAGGCGCGCC GAGGGCCTATTTCCCATGATT
mpknot+AscI-Rev	AATCCGGCGCGCC AACGCCAGCAACGCGGCCTT
PE-sgRNA-AAV-HINDIII-Fw	AGGCAT AAGCTT GCAAGCGATCGCGGGGCCGC
PE-sgRNA-AAV-HINDIII-Rev	AGGCAT AAGCTT GGCGCGCCCACCCTTGGATC
pJet-Fwd	CGACTCACTATAGGGAGAGCGGC
pAAV-Puro-TA-Donor	GCTAACCATGTTCATGCCTTCTTC
AAVS1-Fw	CCCCTTACCTCTCTAGTCTGTGC
AAVS1-Rev	CTCAGGTTCTGGGAGAGGGTAG
hLMNA-geno-fw	CGAGTCTGAAGAGGTGGTCA
hLMNA-geno-rev	GGCAAATCCCAATCTGACCG
RBM20-geno-fw	AGGAGGAGAGTCAGAGGTCC
RBM20-geno-rev	TTTGGGCTCTTTCCGGTAGT
RBM20-geno-Illumina-fw	TGAGTAAAGGCACAGCGAGT
RBM20-geno-Illumina-rev	TGTCCCTCTTGTCATCTCCG
CAG2-fw	TTCGGCTTCTGGCGTGTGACC
bpA-rev	TAGAAGGCACAGTCGAGG
hCAMK2D_E12_fwd	AAGGGTGCCATCTTGACAAC

hCAMK2D_E16_rev	TGCTTTCGTGCTTTCACATC
PE_OT_01_fw	ACCTCTGCCCTCTGATCTGT
PE_OT_01_rev	AGGTCGCTTGCCTTCGTTTA
PE_OT_02_fw	CGGTGCTGTGACTGAGACAT
PE_OT_02_rev	ACAGCCCTGACTACTGTCCT
PE_OT_03_fw	GGAAGGACTCCCTGTCTCCT
PE_OT_03_rev	TCTGGAGAGAAGACTGGGCA
PE_OT_04_fw	GTTGACGGTAGCTGCTTCCT
PE_OT_04_rev	GCGTTTATGAGGGTGAGGCA
PE_OT_05_fw	TGACTGGGCTGCAGAGTTTT
PE_OT_05_rev	GCTGTAAACGTGTGATCGCC

Table S3 sgRNAs

**Name**	**Sequence (5‘>3‘)**
hRBM20-PE3b-ngRNA-1	TACGAGACCGCGGCCTTTCT
hRBM20-PE3b-ngRNA-2	GATATGGCCCAGAAAGGCCG
hRBM20-PE3b-ngRNA-3	TCACCGGACTACGAGACcG
hLMNA-PE3-ngRNA-1	CTGCAGGCGGGCGCGCTCCT
hLMNA-PE3-ngRNA-2	TACTGAGTCAAGGGTCTTGC
AAVS1-sgRNA	CTCAGGTTCTGGGAGAGGGTAG

Supplemental Figures

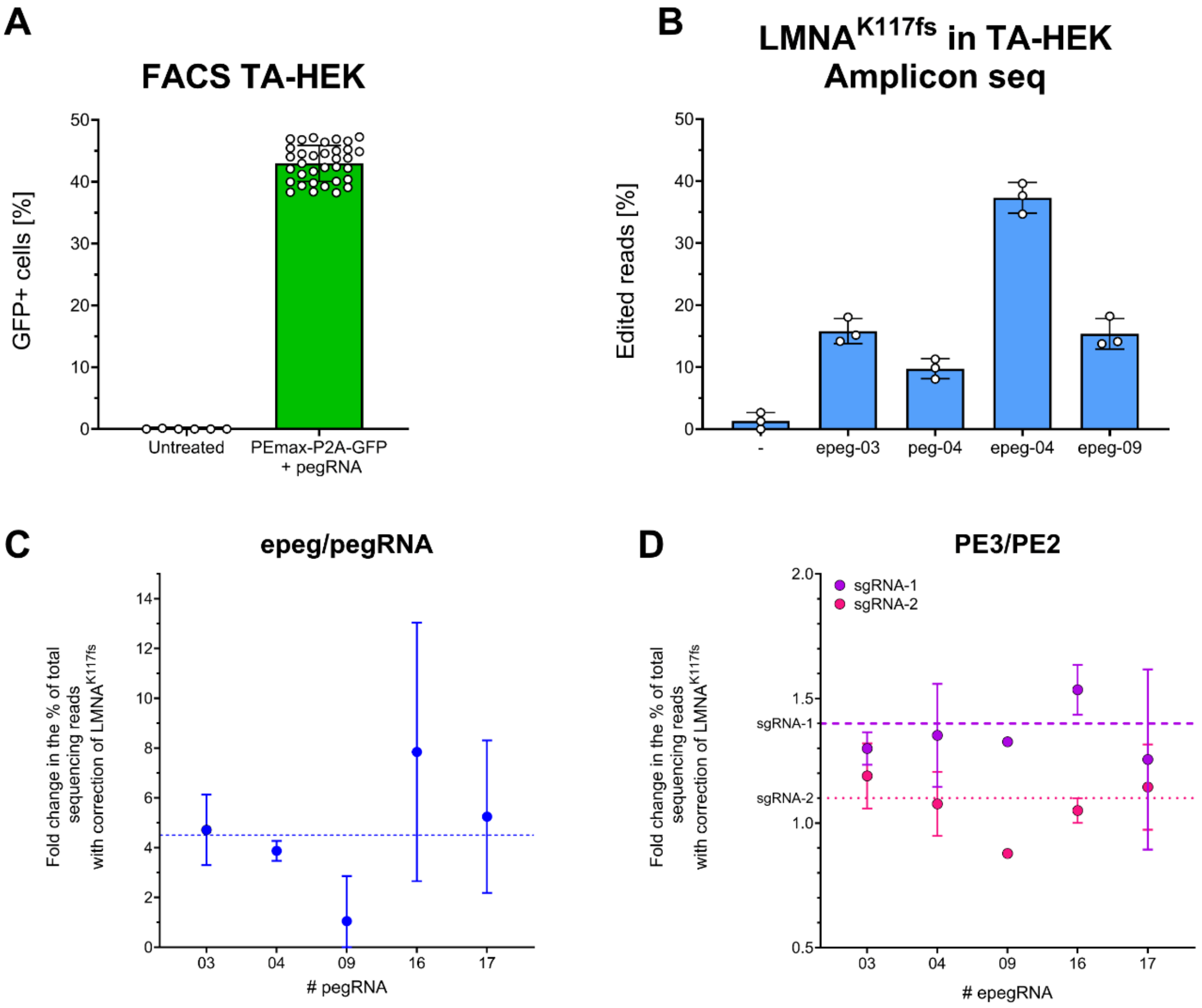

Figure S1: Profiling of pegRNA activity in TA-HEK cells. A Quantification of GFP+ TA-HEK-cells three days after co-transfection with PEmax-p2A-GFP-plasmid and a pegRNA analysed by FACS. Data are expressed as mean (SD; Untreated, n=6; PEmax-p2A-GFP+ pegRNA, n=34 independent experiments). **B** Amplicon sequencing of TA-HEK-cells treated with PE4 and one of five LMNA-(e)pegRNAs. All data are expressed as mean (SD; n=3 independent experiments). **C** Comparison of LMNA-editing efficiency when using epegRNA versus the corresponding pegRNA. Dotted line represents average

fold change across all epegRNAs tested. **D** Comparison of editing efficiency when using the PE2 (no nicking sgRNA) versus PE3 system with the nicking sgRNA-1 or -2. Dashed and dotted line represent average fold change across all LMNA-epegRNAs with sgRNA-1 or -2, respectively. C, D Each dot represents the mean value (SD) of n=3 independent experiments.

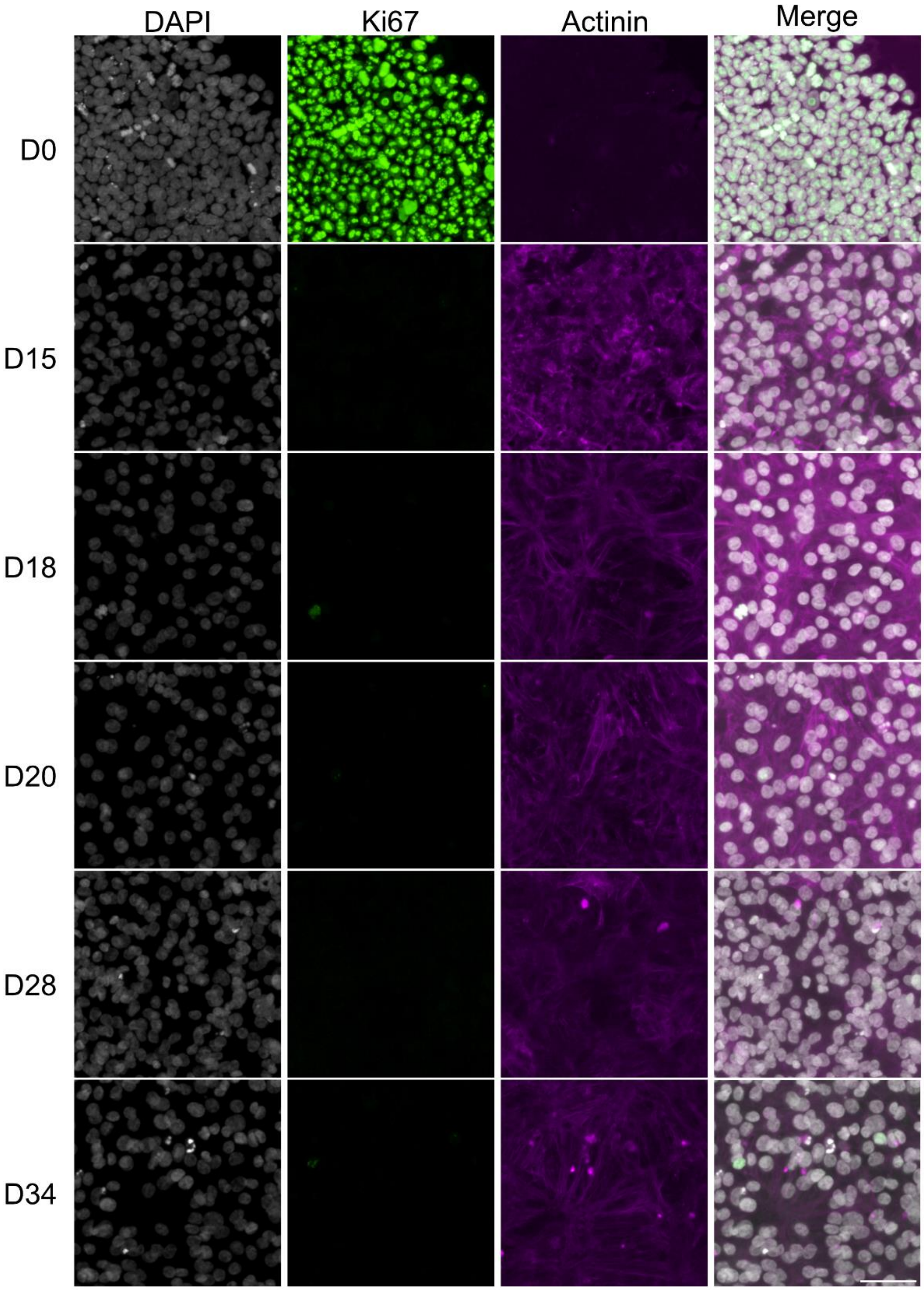
DAPI
Ki67
Actinin
Merge
D0
D15
D18
D20
D28
D34

Figure S2: Immunocytochemistry staining of proliferation and cardiac marker in hi-CMs. Representative images of homozygous RBM20^{P633L} iPSCs (day 0) and hi-CMs at day 15, 18, 20, 28 and 34. Cells were stained for DAPI (grey), Ki67 (green) and α-Actinin (magenta). Scale bar, 50 µm.

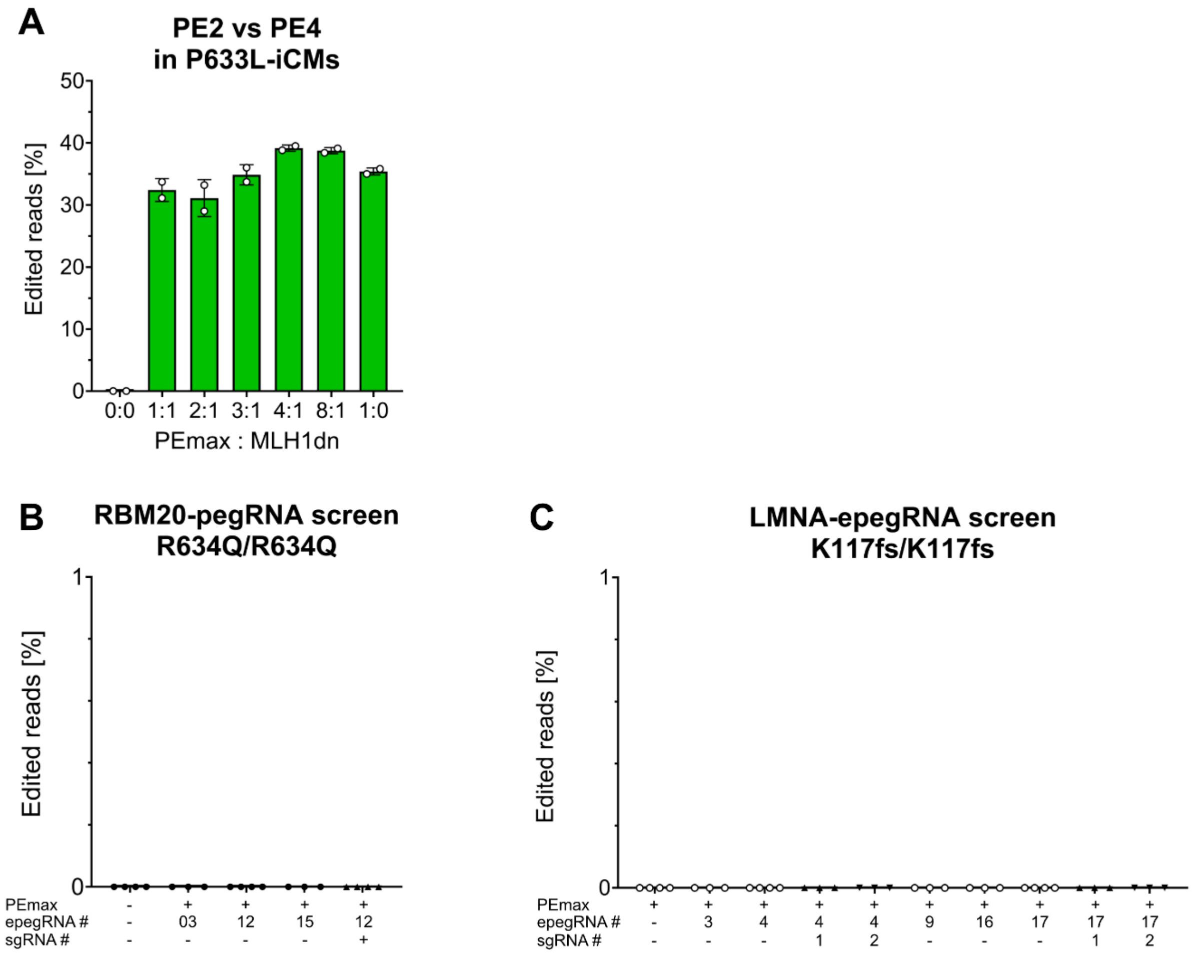

Figure S3: Optimization and epegRNA-screenings in hi-CMs. A Comparison of PE2 versus PE4 editing strategy in homozygous RBM20^{P633L} hi-CMs with epegRNA-12 (n=2 technical replicates). **B** Percentage of A-to-G editing after PE with PEmax and respective epegRNA in hi-CMs carrying the homozygous RBM20^{R634Q}-mutation. Untreated, epegRNA-12, epegRNA-12+sgRNA, n=4 independent experiments. **C** Percentage of edited reads in homozygous LMNA^{K117fs}-hi-CMs after treatment with PE4 or PE5 and indicated epegRNAs. Untreated, epegRNA-04 and -17, n=4 independent experiments. Data are expressed as mean (SD). Three independent experiments (n=3) were performed

unless indicated otherwise. Editing efficiencies were quantified by Sanger sequencing trace deconvolution.

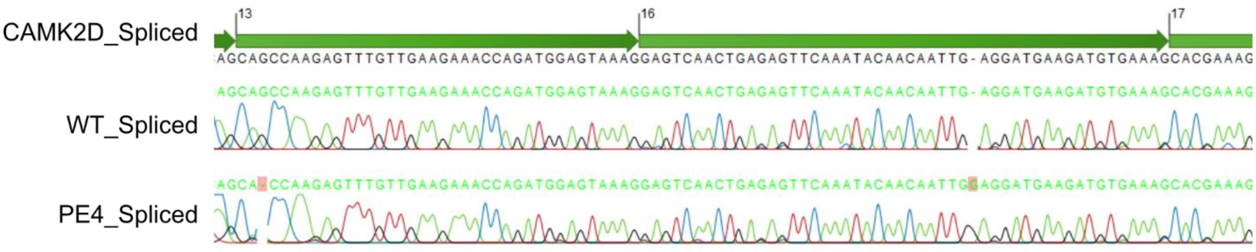

Figure S4: Sequencing validation of the correctly spliced CAMK2D isoform in hi-CMs. Sanger sequencing electropherograms of the wildtype and PE-treated mutant hi-CMs are shown. Exons 13, 16 and 17 are annotated in green.

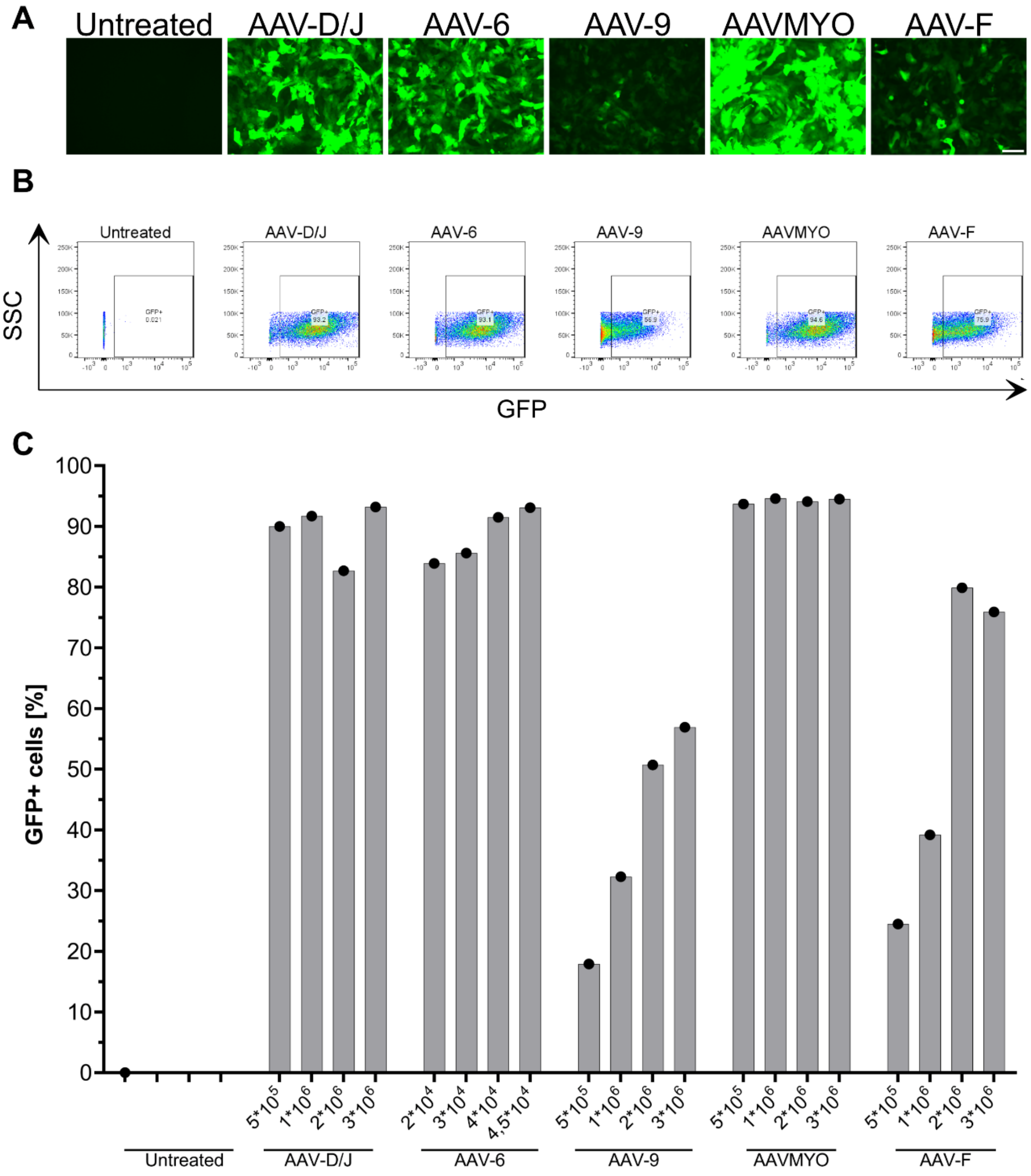

Figure S5: **Quantification of rAAV-transduction efficiencies in hi-CMs with different serotypes. A** Representative fluorescence images of day-21 hi-CMs transduced with GFP-expressing rAAV with respective capsid serotype. Scale bar, 50 µm. **B** Representative flow-cytometry plots showing of GFP+ hi-CMs for each rAAV-serotype. **C**

Quantification of transduction efficiency (% GFP+ cells) by FACS across rAAV-serotypes (n=1 experiment). Level of transduction was quantified as vector genomes per cell (vg/cell).
