# Supplementary material for: Prime editing corrects the dilated cardiomyopathy causing RBM20-P633L-mutation in human cardiomyocytes
Source: Mol Ther Nucleic Acids. 2025 Oct 6;36(4):102734. doi: 10.1016/j.omtn.2025.102734 (PMC12590231; doi:10.1016/j.omtn.2025.102734)
Supplement: Document S2. Article plus supplemental information [file mmc2.pdf]

Molecular Therapy
Nucleic Acids
Original Article

Prime editing corrects the dilated cardiomyopathy causing RBM20-P633L-mutation in human cardiomyocytes

Alexandra Roman,[1,2] Anja Zimmer,[1] Michael Gotthardt,[1,3,4] Lars M. Steinmetz,[5,6,7,8] Ralf Kühn,[1] and Tu Dang[1]

[1]Max Delbrück Center for Molecular Medicine in the Helmholtz Association, Robert-Roessle-Strasse 10, 13125 Berlin, Germany; [2]Freie Universität Berlin, Department of Biology, Chemistry and Pharmacy, Arnimallee 22, 14195 Berlin, Germany; [3]Department of Cardiology, Charité-Universitätsmedizin Berlin, Hessische Strasse 3-4, 10115 Berlin, Germany; [4]DZHK (German Centre for Cardiovascular Research), Partner Site Berlin, Berlin, Germany; [5]Genome Biology Unit, European Molecular Biology Laboratory (EMBL), Heidelberg, Germany; [6]DZHK (German Centre for Cardiovascular Research), Partner Site Heidelberg/Mannheim, Heidelberg, Germany; [7]Department of Genetics, Stanford University School of Medicine, Stanford, CA, USA; [8]Stanford Genome Technology Center, Palo Alto, CA, USA

Prime editing (PE) is an innovative next-generation gene editing tool that has therapeutic potential in post-mitotic organs, such as the human heart. However, its applicability and efficiency in non-proliferating cells, e.g., human cardiomyocytes, is not yet established. Here, we apply PE directly in cardiomyocytes differentiated from human induced pluripotent stem cells (hi-CMs) carrying dilated-cardiomyopathy-causing mutations. A target array (TA) containing the mutations LMNA^{K117fs} (348–349insG), RBM20^{P633L} (c.1898 C>T), and RBM20^{R634Q} (c.1901 G>A) in the safe-harbor locus AAVS1 in HEK293T cells served as a screening platform for prime editing gRNAs (pegRNAs). The pegRNA screen yielded a set of efficient pegRNAs targeting the respective mutations. Using the PE4 system to correct the RBM20^{P633L}-mutation, we achieved 34.8% T-to-C editing efficiency on average in homozygous P633L/P633L-hi-CMs while maintaining low off-target editing. PE restored RBM20's nuclear localization and normalized cardiac splicing of the calcium-/calmodulin-dependent protein kinase II delta (CAMK2D) transcript. We combine a detailed pegRNA screening assay in an easy-to-transfect HEK293T system (TA-HEK) with subsequent functional validation of PE in hi-CMs carrying patient-derived mutations. This strategy yielded the first PE-mediated phenotypic rescue in a human post-mitotic model of DCM and paves the way for an *in vivo* strategy to treat RBM20^{P633L}-mediated DCM and other inherited cardiac diseases.

INTRODUCTION

Prime editing (PE) is a CRISPR-based strategy for introducing a variety of short-sequence changes into the genome, such as insertions, deletions, or substitutions, without creating a double-strand break (DSB) or requiring a separate DNA donor template.[1] The editing complex consists of a Cas9 nickase (nCas9) fused to an engineered reverse transcriptase (RT) and is guided by a specially designed prime editor guide RNA (pegRNA). The pegRNA contains three functional elements: a 20-nucleotide spacer that directs target binding, a primer binding site (PBS) that anneals to the nicked DNA strand, and an RT template (RTT) encoding the desired edit. nCas9 introduces a single-strand nick upon recognition of the target locus, generating a 3′ single-stranded "flap." The PBS of the pegRNA hybridizes to the flap, positioning the RT to reverse transcribe the RTT including the programmed edit into the genome. The process is not restricted to the S and G2 phase of the cell cycle, hence PE can be in principle applied in non-dividing cells.

Post-mitotic organs such as brain and heart are therefore attractive targets for PE-based therapies as their non-proliferative nature renders them impervious to currently established gene editing strategies. The human heart in particular is affected by numerous severe hereditary disorders, among which dilated cardiomyopathy (DCM) has an especially high prevalence. DCM is characterized by an enlarged left ventricle with reduced systolic function and ejection fraction, leading to arrhythmia and progressive heart failure.[2] Roughly 1 out of 220 people are affected by DCM worldwide, of which about 25%–35% of cases are attributable to pathogenic germline variants.[3–5] The most frequently mutated genes in familial DCM are lamin A/C (LMNA) and RNA binding motif 20 (RBM20), accounting for approximately 5.9% and 3% of patients, respectively.[6,7]

Among the editing technologies currently applied in cardiac models, base editors have efficiently corrected cardiomyopathy causing mutations.[8–13] Nevertheless, base editing is limited to specific single-nucleotide conversions within a narrow editing window and cannot address transitions beyond that, nor insertions, deletions, duplications, transversions, or larger variants. Thus, over 2,600 known hereditary cardiac disease mutations caused by short insertions,

Received 4 July 2025; accepted 1 October 2025;
https://doi.org/10.1016/j.omtn.2025.102734.

Correspondence: Ralf Kühn, Max Delbrück Center for Molecular Medicine in the Helmholtz Association, Robert-Roessle-Strasse 10, 13125 Berlin, Germany.
E-mail: ralf.kuehn@mdc-berlin.de
Correspondence: Tu Dang, Max Delbrück Center for Molecular Medicine in the Helmholtz Association, Robert-Roessle-Strasse 10, 13125 Berlin, Germany.
E-mail: tu.dangngoc@mdc-berlin.de

Published by Elsevier Inc. on behalf of The American Society of Gene and Cell Therapy.
This is an open access article under the CC BY license (http://creativecommons.org/licenses/by/4.0/).

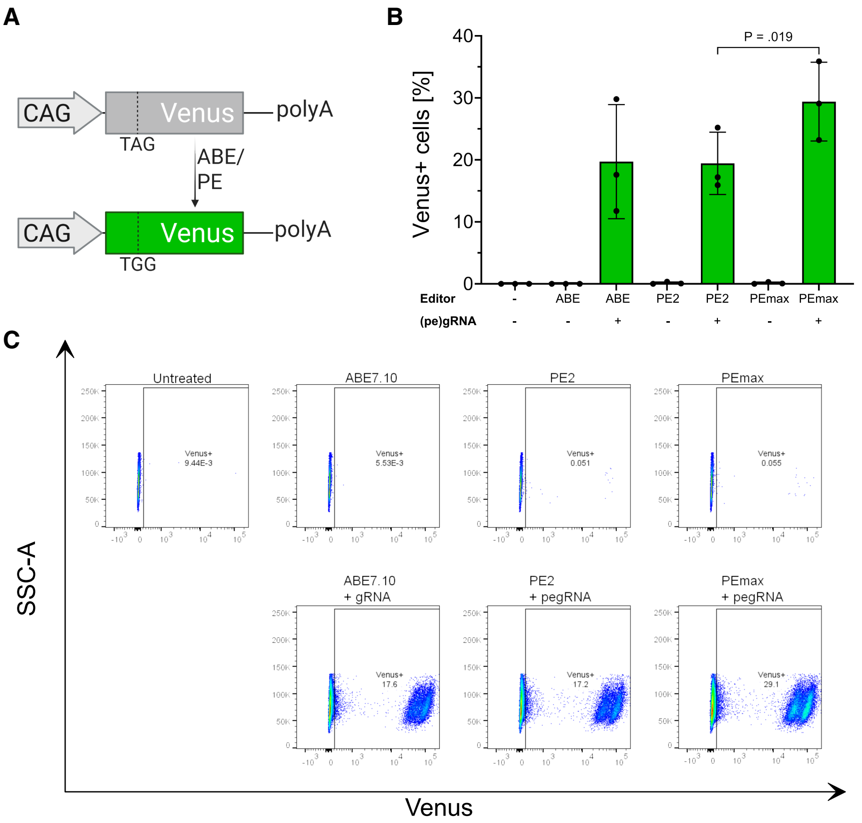

Figure 1. Validation of PEmax performance in Venus-reporter HEK cells

(A) Schematic illustration of ABE/PE reporter system in Venus-reporter HEK cells. (B and C) FACS quantification of Venus-reporter HEK cells after base (ABE7.10) and prime editing (PE2 and PEmax). Data are expressed as mean (SD) from three biological replicates (n = 3 independent experiments). Paired, two-tailed Student's t test was performed.

deletions, indels, and duplications fall outside the scope of current base editing chemistries.[14] PE, on the other hand, has the potential to target the entire spectrum of these mutations, making it a versatile platform for precision gene editing in the heart.[15]

In earlier studies, PE has been shown to work in the mouse heart, reaching up to 11% editing efficiency of a benchmark C-to-G substitution in the Dnmt1 locus.[16] Although encouraging, murine results do not necessarily predict the therapeutic potential of PE in human cardiomyocytes. Nishiyama et al. used PE to correct the R636S (c.1907 G>T) mutation in the RBM20 gene in proliferating human induced pluripotent stem cells (iPSCs).[8] Similarly, Chemello et al. applied a PE3 strategy to introduce a +2-nucleotide insertion in exon 52 of the dystrophin (DMD) gene of ΔEx51-iPSCs, which restored dystrophin expression and contractile function after differentiation.[17] However, both studies relied on editing in dividing iPSCs prior to cardiac differentiation, leaving PE's efficiency and safety in nondividing, post-mitotic human cardiomyocytes still unknown.

Wang et al. extended PE to human-iPSC-derived cardiomyocytes (hi-CMs) carrying a deletion spanning exons 45–50 in the DMD gene (DMD.ΔEx45-50).[18] This mutation disrupts the DMD reading frame and predominantly causes skeletal muscle dystrophy. By inserting a single nucleotide in exon 51, Wang et al. restored the reading frame and expression of truncated dystrophin (Becker-like dystrophin). However, the strategy did not correct the gene precisely or restore the wildtype (WT) phenotype in hi-CMs.

In this study, our goal is to demonstrate that PE can precisely repair patient-derived DCM mutations to wildtype in differentiated, non-dividing human cardiomyocytes. We focused on three pathogenic DCM-causing mutations within the human LMNA and RBM20 genes. First, an allele-specific PE strategy was implemented to target the heterozygous $LMNA^{K117fs}$ frameshift (348–349insG) mutation. In addition, we designed a PE approach to correct both the $RBM20^{P633L}$ (c.1898 C>T) and the $RBM20^{R634Q}$ (c.1901 G>A) mutation concurrently. To streamline guide optimization, we developed a modular system for pegRNA screening using transfectable HEK293T cells (TA-HEK), followed by functional validation in post-mitotic hi-CMs. Using this approach, we precisely and efficiently repaired the $RBM20^{P633L}$ mutation in hi-CMs, restored RBM20 nuclear localization, and normalized calcium-/calmodulin-dependent protein kinase II delta (CAMK2D) splicing. These findings provide the first demonstration that PE can efficiently correct disease-causing DCM mutations and rescue downstream molecular defects in a human post-mitotic cardiac model. Given the robust rescue and minimal off-target activity, our results support further pre-clinical development of PE-based therapies, particularly for $RBM20^{P633L}$-associated DCM.

RESULTS

Validation of PE2 and PEmax editing efficiency in a Venus-reporter HEK cell line

To initially validate the functionality of prime editors, we conducted benchmark experiments in fluorescence reporter HEK293T cells (Venus-reporter cells). In this reporter system, a mutated Venus coding sequence is stably integrated into the AAVS1 safe-harbor locus and driven by the constitutive CAG promoter. A premature stop-codon (TAG) in the coding sequence of Venus abolishes its expression. Successful installation of an A-to-G-edit in the stop codon (TAG-to-TGG) restores Venus expression, indicating correct base substitution (Figure 1A).

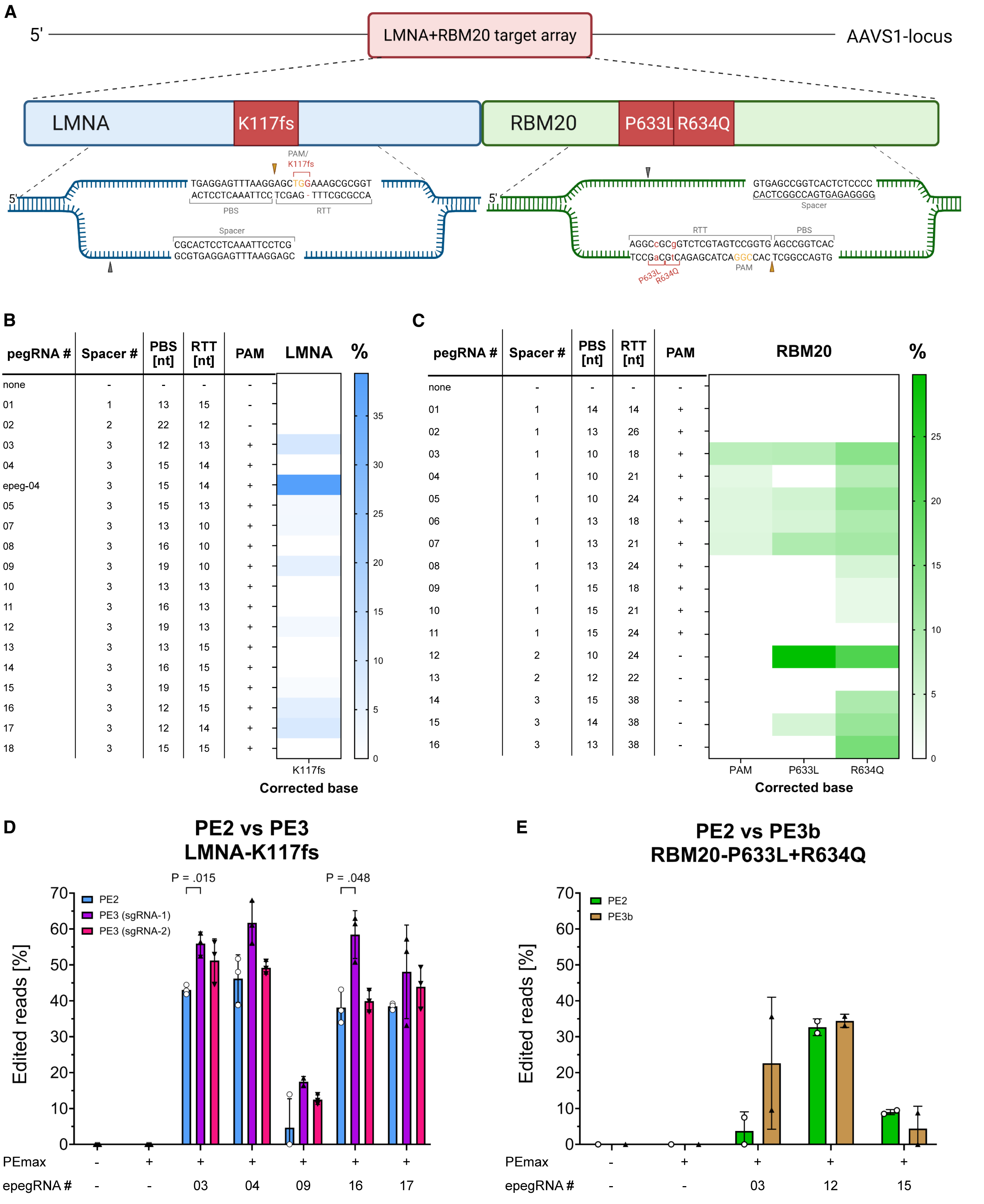

(legend on next page)

We compared the canonical PE2 editor with the enhanced PEmax variant.[19] Each editor was fused to a P2A-GFP tag and co-transfected with a plasmid expressing a pegRNA. An adenosine base editor (ABE7.10) served as a positive control. Transfected cells were cultured for 3 weeks before analysis by flow cytometry, long enough for transient GFP expression from the P2A-GFP tag to dissipate (Figures 1B and 1C). ABE7.10 yielded 19.7% ± 9.2% Venus positive cells; 19.4% ± 5% of the cells expressed Venus after editing with PE2, which could be increased to 29.4% ± 6.3% using PEmax (Figure 1B). Based on its superior editing efficiency compared to PE2, PEmax was selected for further experiments.

pegRNA screening in target array cell line identifies high-performing candidates for repairing DCM-causing mutations

We selected three DCM-related mutations as PE targets in hi-CMs due to their clinical relevance: the $LMNA^{K117fs}$ frameshift (c.348_349insG) and two missense variants in the RBM20 gene, $RBM20^{P633L}$ (c.1898 C>T) and $RBM20^{R634Q}$ (c.1901 G>A).[20–22] $LMNA^{K117fs}$ describes a guanine insertion in the first exon of the LMNA gene, which generates a premature stop codon, leading to Lamin A/C haploinsufficiency and progressive DCM.[21] Due to the two substitutions $RBM20^{P633L}$ and $RBM20^{R634Q}$ within the RSRSP-stretch in exon 9, RBM20 is mislocalized to the cytoplasm, aggregates into stress granules, and subsequently leads to aberrant splicing of cardiac genes such as Titin and CAMK2D.[20,23–28]

pegRNAs were selected using web tools such as PrimeDesign, pegFinder, pegIT, and pridict.it or designed manually according to the results from previous publications.[29–35] For each locus, we chose three independent spacer sequences and paired them with varying 3′-extensions, yielding 17 candidate pegRNAs targeting the LMNA locus and 16 targeting the P633L and R634Q mutations in the RBM20 locus.

The guanine insertion in the $LMNA^{K117fs}$ mutation generates a new NGG-PAM, permitting mutant-allele-specific editing in heterozygous cells (Figure 2A). This feature is exploited in LMNA-pegRNAs #3–18. RBM20-pegRNAs #1–11 were designed to introduce a silent PAM-disrupting mutation to prevent re-targeting of corrected alleles. The targeting mechanism of two representative guides, LMNA-pegRNA-04 and RBM20-pegRNA-12, are shown in Figure 2A. All pegRNAs were initially cloned into the generic backbone according to Doman *et al.*[33] LMNA-pegRNA-04 was additionally cloned into the engineered pegRNA (epegRNA) backbone with the pseudoknot from Moloney murine leukemia virus (mpknot) to stabilize the 3′ extension (designated LMNA-epeg-04).[36]

A target-array-HEK293T cell line (TA-HEK) was generated to screen a high number of pegRNAs for each locus in an efficient and detailed manner before selecting the best performing pegRNAs for experiments in hi-CMs. To this purpose, a 156 bp fragment of LMNA carrying the K117fs insertion and a 174 bp stretch of RBM20 harboring the P633L and R634Q substitutions were concatenated and stably integrated into the AAVS1 locus (Figure 2A). TA-HEK cells were co-transfected with PEmax-P2A-GFP and the respective pegRNA plasmid. After 72 h, an average of 43% ± 2.9% of the transfected cells were GFP-positive and were sorted from the bulk population via FACS (Figure S1A). Genomic DNA was extracted from sorted cells and analyzed by Sanger sequencing to evaluate the PE efficiency of each pegRNA. Sanger traces identified robust K117fs correction with LMNA-pegRNA-03, -09, -16, and -17 and the engineered pegRNA LMNA-epegRNA-04 (Figure 2B). Amplicon sequencing confirmed that LMNA-epegRNA-04 provided the highest editing efficiency (37.3% ± 2.5%), followed by LMNA-pegRNA-03 (15.8% ± 2.0%) and LMNA-pegRNA-09 (15.4% ± 2.5%) (Figure S1B). Because LMNA-epegRNA-04 outperformed its generic-backbone counterpart (9.8% ± 1.6%), we recloned LMNA-pegRNA-03/-09/-16 and -17 into the same mpknot backbone. This backbone modification boosted editing 4.5-fold on average (Figure S1C). Notably, PE with LMNA-epegRNA-16 achieved a 38.1% ± 4.7% editing efficiency as compared to LMNA-pegRNA-16 with 6.4% ± 3.7%, reaching a 7.8-fold improvement (Figures 2D and S1C).

Among the 16 pegRNAs tested for targeting the RBM20 locus in TA-HEK cells, six pegRNAs simultaneously edited the PAM (if applicable), P633L, and R634Q mutation. Highest editing efficiency was achieved when using RBM20-pegRNA-12, correcting 29.8% ± 7.2% of P633L alleles and 20.5% ± 1.5% of R634Q (Figure 2C). Based on the positive results from recloning the LMNA-pegRNAs into the engineered pegRNA backbone with the mpknot, one representative guide from each spacer group (RBM20-pegRNA-03, -12, and -15) was tested as epegRNA. The resulting epegRNAs (RBM20-epegRNA-03/-12 and -15) produced 3.8% ± 5.3%, 32.7% ± 2.3%, and 9.0% ± 0.7% editing rates across both mutations and the PAM, if applicable (Figure 2E).

After choosing the best-performing epegRNAs for each locus, we sought to further enhance PE by adding a nicking single guide RNA (sgRNA) as described in the PE3(b) system.[1] Each

Figure 2. pegRNA screen for correcting LMNA and RBM20 mutations in target-array-HEK cells

(A) Schematic representation of the target array (TA) in HEK293T cells harboring the $LMNA^{K117fs}$, $RBM20^{P633L}$, and $RBM20^{R634Q}$ mutation. Prime editing strategy with LMNA-pegRNA-4 and RBM20-pegRNA-12 is shown as example. The brown and gray arrowheads indicate the nicking site of the pegRNA and sgRNA, respectively. (B and C) Screening results for targeting $LMNA^{K117fs}$ (B) and $RBM20^{P633L}$ and $RBM20^{R634Q}$ mutations (C) in TA-HEK cells. Heatmap shows the editing efficiency (%) of each pegRNA measured by Sanger sequencing trace deconvolution. PBS, primer binding site; RTT, reverse transcriptase template. PAM+ pegRNAs disrupt the PAM. Data represent mean values from three biological replicates (n = 3 independent experiments). (D and E) Comparison of PE2 (one nick) versus PE3 (with second nicking sgRNA) using selected epegRNAs targeting the LMNA (D) and the RBM20 (E) locus. Editing efficiencies are quantified by Sanger sequencing trace deconvolution and are shown as mean (SD; n = 3 in D and n = 2 independent experiments in E) for the specified edits. Statistical significance was evaluated by an unpaired two-tailed Student's t test.

LMNA-epegRNA was tested in combination with either sgRNA-1 or -2 in the frame of the PE3 system. sgRNA-1 consistently outperformed sgRNA-2, increasing editing on average 1.4-fold versus 1.1-fold (Figure S1D). For example, editing efficiency increased from 38.1% ± 4.6% to 58.4% ± 6.6% when using epegRNA-16 with sgRNA-1, generating a 1.5-fold improvement (Figures 2D and S1D). The RBM20 epegRNAs responded heterogeneously in the PE3b system: PE with RBM20-epegRNA-03 improved 6-fold, remained unchanged with RBM20-epegRNA-12, and decreased slightly with RBM20-epegRNA-15 (Figure 2E).

pegRNA screens in TA-HEK cells showed that PE can efficiently correct the LMNA^{K117fs}, RBM20^{P633L}, and RBM20^{R634Q} mutations to wildtype sequence and provide a pre-selection of efficient (e)pegRNAs for correcting patient-derived mutations. Editing results can be optimized by implementing epegRNAs and a second nicking gRNA when targeting the LMNA locus. Altogether, embedding and targeting clinically relevant mutations in an accessible locus in highly transfectable HEK293T cells accelerates and facilitates the process of pegRNA optimization and selection before proceeding to high-maintenance and hard-to-transfect cells.

Prime editing corrects the RBM20^{P633L} mutation in post-mitotic hi-CMs

Using the best-performing epegRNAs identified in the TA-HEK screen, we subsequently established the PE strategy in hi-CMs. iPSCs carrying either patient mutation were differentiated to hi-CMs, which were transduced on day 21 of differentiation with a recombinant AAV vector (rAAV-D/J) carrying an epegRNA. Synthetic mRNAs for both components of the PE4 system, the PEmax editor and the mismatch mediated repair (MMR) inhibitor MLH1dn, were cotransfected into the same cells 24 h later. Genomic DNA was harvested 5 days after mRNA delivery. Editing efficiency was quantified in bulk hi-CM cultures by Sanger or amplicon sequencing (Figure 3A).

To verify that our target cells were post-mitotic, we performed immunocytochemistry staining of the proliferation marker Ki-67 and the sarcomeric marker α-actinin at different days of differentiation. By day 15, Ki-67 was absent in the differentiated hi-CMs, whereas α-actinin was strongly expressed, confirming a non-proliferative cardiomyocytic phenotype (Figure S2).

MMR is known to impede PE but can be inhibited by transiently expressing MLH1dn, constituting the PE4 system.[19] Although the hi-CMs exhibit no or very little proliferative activity and are likely to have no DNA-MMR,[37] different ratios of PEmax and MLH1dn-mRNA were tested in hi-CMs to exclude inhibition of PE through residual MMR activity. Using RBM20-epegRNA-12 in hi-CMs with the homozygous RBM20^{P633L} (P633L/P633L) mutation, T-to-C editing without MLH1dn-mRNA (PE2-system) was detected in 35.4% ± 0.6% of reads (Figure S3A; PEmax:MLH1dn ratio = 1:0). The editing efficiency increased to 39.2% ± 0.5% when adding MLH1dn-mRNA in a PEmax:MLH1dn-ratio of 4:1 (Figure S3A), indicating a positive effect of MMR inhibition even in post-mitotic hi-CMs. This effect can be presumably attributed to the immature state of hi-CMs or remaining activity of MLH1 in hi-CMs.[37,38] According to these results, the PE4 system with a PEmax:MLH1dn-ratio of 4:1 will be used in further experiments.

RBM20-epegRNA-03/-12 and -15, identified as the most efficient guides in the TA-HEK screen, were evaluated further in hi-CMs with the homozygous RBM20^{P633L} mutation. Surprisingly, only epegRNA-12 produced detectable editing, achieving 34.8% ± 4.6% T-to-C conversion with the PE4 system (Figure 3B).

Addition of a nicking sgRNA (PE5 system) to hi-CMs yielded inconsistent results, reaching a maximum editing efficiency of only 25.8% (Figure 3C). Hence, we used the PE4 system further on to minimize the risk of indels and off-target editing associated with a second nick. Amplicon sequencing of homozygous P633L-hi-CMs targeted with epegRNA-12 in the PE4 system confirmed the previous results by showing 29.2% ± 5.6% of precise T-to-C conversion, with low indel (3.7% ± 1.5%) and scaffold integration (0.2% ± 0.05%) frequency (Figure 3D). Since patients carry a heterozygous mutation, the PE4 strategy was tested in isogenic hi-CMs with the heterozygous RBM20^{P633L} mutation (WT/P633L). We observed 65.6% ± 1.4% of WT reads in treated samples, reaching a significant increase compared to untreated heterozygous samples (with an observed 49.1% of WT alleles, $p < .001$, Figure 3E). To analyze the off-target (OT) effects of RBM20-epegRNA-12, we employed amplicon sequencing analysis of the five highest-scoring *in-silico*-predicted OT sites. The indel frequency at these sites did not differ between the treated hi-CMs and the untreated controls, indicating that epegRNA-12 exhibits high target specificity (Figure 3F).

Since the RBM20 editing strategy was designed to target both the P633L and R634Q mutations, we tested RBM20-epegRNA-12 in hi-CMs with the homozygous RBM20^{R634Q} allele (R634Q/R634Q) under the same PE4 conditions. PE4 resulted in 4.6% ± 0.6% of A-to-G conversion in hi-CMs with 2.2% ± 0.6% indel frequency and 0.08% ± 0.03% scaffold incorporation (Figure 3G). Adding the nicking sgRNA did not improve the outcome, and none of the other top-performing pegRNAs, RBM20-epegRNA-03 or -15, produced detectable desired editing with PE4 (Figure S3B).

PE of the LMNA^{K117fs} mutation with LMNA-epegRNA-03, -04, -09, -16, and -17 yielded high correction levels in TA-HEK cells and was subsequently tested in hi-CMs with the homozygous LMNA^{K117fs} mutation (K117fs/K117fs). Sanger sequencing detected no correction in treated cells with any of the epegRNAs, neither the PE4 nor the PE5 system (Figure S3C). However, amplicon sequencing revealed correct editing with LMNA-epegRNA-04 in 1.05% ± 0.09% of reads and with LMNA-epegRNA-17 in 1.12% ± 0.30% of reads. The editing rate is rather low but significantly higher compared to control samples (untreated). Indels and scaffold incorporation were negligible with both epegRNAs (<0.02% ± 0.02% and 1×10^{-5}% ± 1.7×10^{-5}%, respectively; Figure 3H).

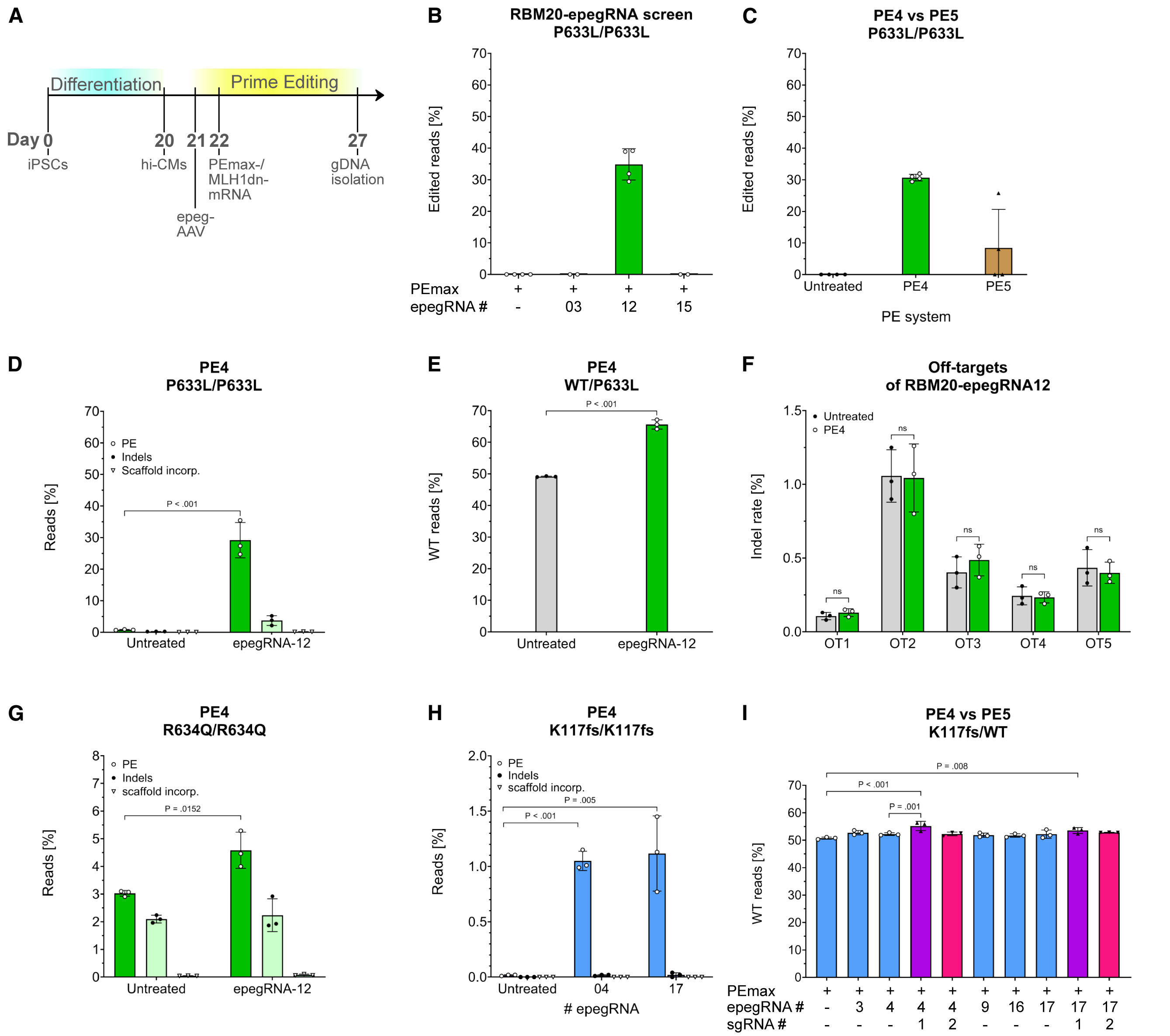

Figure 3. Evaluation of PE targeting LMNA and RBM20 in hi-CMs

(A) Experimental timeline of PE in hi-CMs. (B) Percentage of T-to-C editing after PE4 editing with PEmax and respective epegRNA in hi-CMs carrying the homozygous RBM20^{P633L} mutation. W/o, *n* = 4; epegRNA-03/-15, *n* = 2; epegRNA-12, *n* = 4 independent differentiations. (C) Comparison of editing efficiencies when using the PE4 and the PE5 strategy with epegRNA-12 in homozygous RBM20^{P633L}-hi-CMs (*n* = 4 independent differentiations). (D) Amplicon sequencing of PE4-edited homozygous RBM20^{P633L}-hi-CMs with epegRNA-12. Reads containing the edit, indel frequency, and scaffold incorporation are shown as percentage. (E) Quantification of wild-type reads in PE4-edited heterozygous WT/P633L-hi-CMs with epegRNA-12. (F) Amplicon sequencing of potential off-target (OT) sites of RBM20-epegRNA-12 in homozygous P633L-hi-CMs. (G) Amplicon sequencing of homozygous R634Q-hi-CMs upon editing with PE4 and RBM20-epegRNA-12. Reads containing the desired edit (A-to-G conversion), indels, and scaffold incorporation are shown. (H) Amplicon sequencing of homozygous LMNA^{K117fs}-hi-CMs after treatment with PE4 with LMNA-epegRNA-04 or -17. Reads containing the desired edit (A-to-G conversion), indels, and scaffold incorporation. (I) Quantification of wild-type reads of heterozygous LMNA WT/K117fs-hi-CMs after treatment with PE4 or PE5 and indicated epegRNAs. One-way ANOVA with Dunnett's multiple comparisons test was performed. Editing efficiencies of (B, C, E, and I) were quantified by Sanger sequencing trace deconvolution. All data are expressed as mean (SD; *n* = 3 independent experiments, unless indicated otherwise). (D–H) Unpaired two-tailed Student's t test was performed. ns, no significance.

Because the K117fs mutation creates a unique NGG-PAM, the mutant allele can be corrected selectively in heterozygous cells without targeting the WT allele. In heterozygous hi-CMs (K117fs/WT), the PE5 strategy with sgRNA-1 increased the fraction of WT reads from 50.7% ± 0.4% in untreated cells to 55.2% ± 1.6% and 53.5% ± 1.0% in hi-CMs treated with epegRNAs-04 and -17, respectively (Figure 3I). These gains correspond to approximately 4.5% and 2.8% of corrected mutant alleles, representing a significant improvement over baseline.

Collectively, these data demonstrate that PE can robustly and efficiently repair the patient-derived RBM20^{P633L} mutation in unsorted, post-mitotic hi-CMs while generating only minimal indels and scaffold integration. By contrast, correction of the other two targeted mutations (RBM20^{R634Q} and LMNA^{K117fs}) remains modest in hi-CMs, despite their high editing efficiencies in the TA-HEK screen.

Correction of RBM20^{P633L} restores protein localization and cardiac splicing

After achieving efficient genomic repair of the RBM20^{P633L} mutation in hi-CMs, we proceeded to examine whether the molecular phenotype associated with this mutation was also rescued. As described previously, the P633L mutation within the RS domain of RBM20 disrupts its interaction with Transportin-3 (TNPO3), thus preventing nuclear import and causing cytoplasmic aggregation of the protein.[13,39]

Consistent with the literature, immunocytochemistry staining followed by confocal microscopy showed a diffuse RBM20 expression pattern in homozygous RBM20^{P633L} mutant hi-CMs, with RBM20 being localized equally in the cytoplasm and in the nucleus on day 34 of differentiation (Figures 4A and 4B). In contrast, PE4-edited cells showed a predominantly nuclear RBM20 localization pattern that closely resembled WT control cells (Figure 4A). Quantification of spatial correlation of RBM20 and DAPI supported our findings. PE4-treated and WT hi-CMs yielded a similarly high Pearson coefficient (0.55 ± 0.05 and 0.42 ± 0.07, respectively), indicating colocalization, whereas untreated mutant hi-CMs displayed a significantly lower value (0.2 ± 0.03, p = .001; Figure 4B).

Finally, we tested if correcting the P633L mutation rescues RBM20-dependent cardiac splicing of CAMK2D. CAMK2D is not spliced correctly in the absence of RBM20, which leads to retention of exon 15 in the mature mRNA (Figure 4C). Amplification of the CAMK2D-cDNA with primers spanning exon 12–16 produces a 178 bp large amplicon as can be observed in untreated P633L-hi-CMs. After treating hi-CMs with PE4, a strong lower band at 136 bp can be observed, which corresponds to the correctly spliced short isoform lacking exon 15 (Figures 4C and S4). Densitometric analysis revealed that the fraction of correctly spliced CAMK2D increased 4.1-fold in PE-treated compared to untreated hi-CMs, indicating a decrease in exon 15 expression (Figure 4D). The short isoform constitutes 33.3% ± 13.7% of the total splice-PCR products, on average, 2 weeks after incubation with the prime editor, while only 8.2% ± 2.7% is detected in untreated samples (Figure 4D). The data demonstrate that the ratio of correctly spliced to unspliced CAMK2D shifts toward the short isoform upon correct nuclear localization, rescuing correct cardiac isoform expression.

DISCUSSION

PE is a next-generation gene editing tool capable of installing virtually any small DNA changes without relying on homology-directed repair. This feature is especially attractive for tissues with negligible regenerative capacity, such as the adult human heart. Although initial studies hint that PE could correct DCM mutations, its true therapeutic potential in a post-mitotic human cardiac context remains unknown. Nishiyama et al. used PE to correct a DCM-causing mutation in RBM20 but did not test PE's ability to correct the mutation in post-mitotic human cardiomyocytes.[8] In contrast, Wang et al. have applied PE to hi-CMs carrying a deletion spanning exons 45–50 in the DMD gene, a gene primarily expressed in skeletal muscle rather than in the heart. Wang et al. inserted a single, aberrant nucleotide in exon 51 of the DMD gene to restore the codon reading frame and the expression of truncated Becker-like dystrophin.[18] However, this strategy did not precisely repair the disease-causing mutation or restore the WT protein. This degree of flexibility is not available when targeting the majority of missense single-nucleotide polymorphisms (SNPs), especially DCM causing mutations in RBM20 and LMNA.

In this study, we demonstrate that PE can precisely correct patient-derived DCM mutations to WT in differentiated, non-dividing human cardiomyocytes. We establish a modular workflow for PE-mediated repair of clinically relevant mutations, which couples a detailed pegRNA screening in a highly transfectable HEK293T system (TA-HEK) with subsequent functional validation in hi-CMs. The TA-HEK cell line, harboring a TA consisting of patient-derived mutations at the AAVS1 locus, enables parallel testing and optimization of pegRNA designs. This system is easily adaptable to any prime editor variant or pegRNA structure modification and streamlines plasmid-based prime editor and pegRNA testing without the need for laborious RNA *in vitro* synthesis or rAAV production.

High-performing pegRNAs identified in the TA-HEK screen can be partially transferred to hi-CMs. For instance, RBM20-epegRNA-12 achieved 32.7% correction of the RBM20^{P633L} mutation in TA-HEK cells and 34.8% in hi-CMs carrying the homozygous P633L mutation, restoring correct nuclear localization of RBM20 and rescuing cardiac splice defects with minimal off-target activity. However, our results show that editing efficiencies cannot be generally translated from TA-HEK cells to hi-CMs. PE of the RBM20^{R634Q} mutation in TA-HEK cells corrected 20.5% of transfected cells but performed low in unsorted homozygous hi-CMs (4.6%) with the same pegRNA. We hypothesize one influencing factor to be the varying severity of the locally adjacent RBM20 mutations. Previous confocal imaging in hi-CMs has shown that the RBM20^{P633L} mutation causes only partial cytoplasmic mislocalization of RBM20, whereas RBM20^{R634Q} leads to pronounced aggregation of the protein into cytoplasmic granules.[39] The resulting milder mis-splicing in

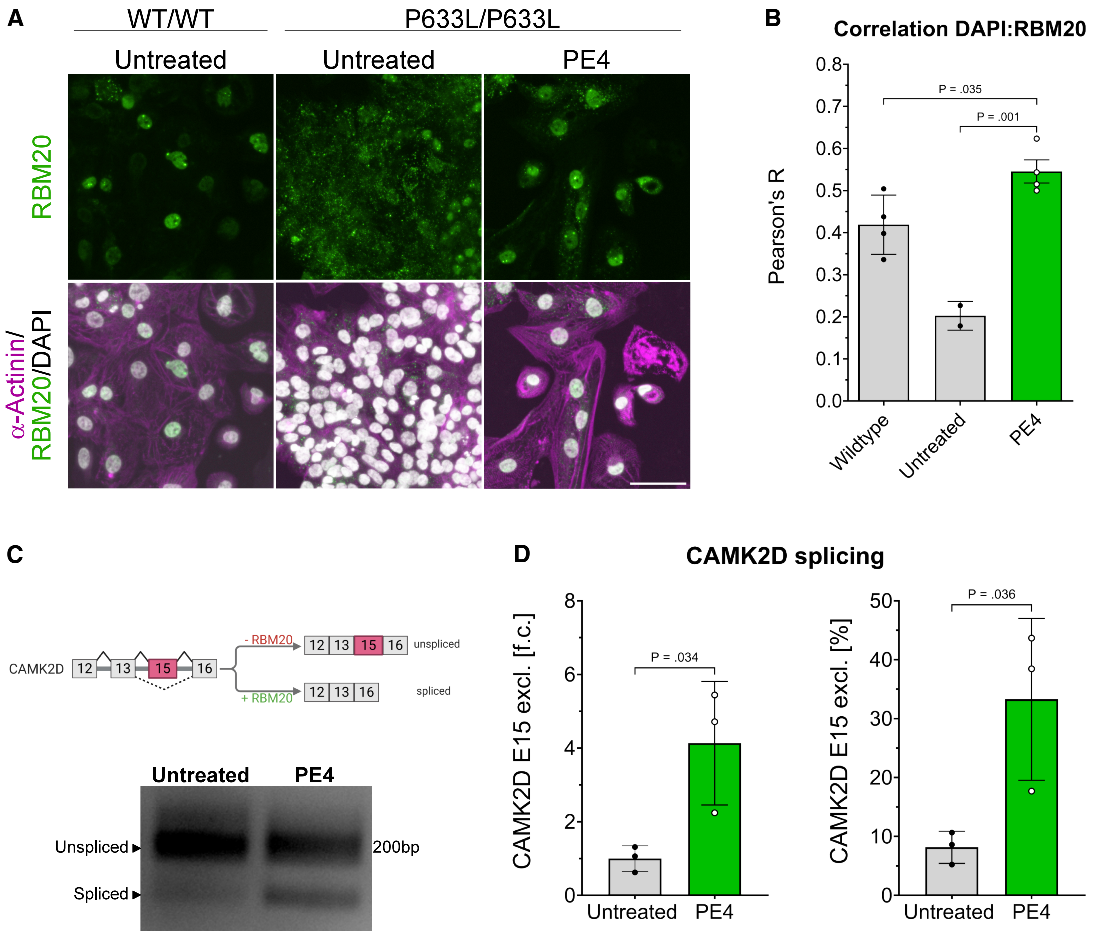

Figure 4. Prime editing of the RBM20^{P633L} mutation in hi-CMs restores RBM20 localization and rescues CAMK2D splicing

(A) Representative immunocytochemistry images of isogenic wild-type (WT/WT), untreated, and PE4-treated homozygous P633L-hi-CMs. DAPI (gray), RBM20 (green), and α-Actinin (magenta). Scale bars, 50 μm. (B) Colocalization analysis of RBM20 with DAPI based on images from (A). Each dot represents the Pearson coefficient R of at least five nuclei. Data are expressed as mean (SD; untreated, *n* = 2; WT and PE4, *n* = 4 technical replicates). (C) Schematic illustration of RBM20-dependent splicing of CAMK2D-mRNA (top). Reverse transcription (RT)-PCR of CAMK2D cDNA using primers spanning exons 12–16 in hi-CMs with indicated treatment (bottom). Representative agarose gel for three biological replicates. (D) Densitometric analysis of CAMK2D RT-PCR products. Ratio of lower-to-total band intensity normalized to the untreated control (left). Percentage of the short CAMK2D isoform relative to total RT-PCR products (right). Data are expressed as mean (SD) of three biological replicates (*n* = 3 independent differentiations). RT-PCR and densitometry were performed 3 to 4 times for each sample. (B and D) Unpaired two-tailed Student's t test was performed. f.c., fold change.

RBM20^{P633L} hi-CMs is assumed to increase cell resilience during viral transduction and mRNA transfection, resulting in higher survival of targeted cells and, thus, a greater correction efficiency. In contrast, the more severe phenotype in RBM20^{R634Q} hi-CMs may sensitize these cells to the delivery-induced stress, reducing the pool of viable, edited cells and yielding lower correction rates as observed.

A similar effect was observed when targeting the LMNA^{K117fs} mutation, as editing rates decreased from 37.3% in TA-HEK cells to 1.05% in homozygous hi-CMs. Lee et al. observed that the LMNA^{K117fs}-induced Lamin A/C haploinsufficiency epigenetically activates the PDGF signaling pathway in hi-CMs.[21] The PDGF signaling activation indicates an increased stress level in mutant hi-CMs, which may render the cells susceptible to transfection- and transduction-related toxicity.

These cell-line- and allele-specific differences should be examined by adjusting the doses of the prime editor and epegRNAs more carefully and by investigating the viability of the cells post-treatment. Future work should also explore truncated, catalytically "dead" sgRNAs, a strategy recently shown to increase the accessibility of prime editors to difficult-to-edit target sites by modulating the chromatin state.[40]

Adding a nicking sgRNA (PE3 and PE5 strategy) increased editing efficiencies slightly in the LMNA locus (Figures 2D and 3I), while the PE3b and PE5b strategy reduced rates at the RBM20^{P633L} mutation in comparison with the no-nick configuration (PE2 and PE4;

Figures 2E and 3C). The PE3b strategy exploits that the intended edit reconstitutes a correction-specific PAM, which is in turn targeted by the nicking sgRNA. Thus, the secondary nick is only introduced in edited alleles. According to the work published by Anzalone et al., the PE3b strategy was favored over the PE3 strategy when targeting the RBM20 mutations, as the nicking sgRNA matches the edited strand only after correction of both RBM20 mutations. Notably, correction of the R634Q mutation reconstitutes the PAM targeted by the sgRNA (Figure 2A). However, the PE3b strategy did not enhance but reduced editing rates, suggesting a steric hindrance of both sgRNAs and pegRNAs at the edit site due to the close proximity of the two nicking sites (21 bp). A temporal separation of sgRNA and pegRNA delivery when using the PE3b strategy or testing the PE3 strategy with greater distances between the two nicks should be investigated in future studies.

Previous work by Grosch et al. and Nishiyama et al. have successfully used base editing to correct the RBM20^{P633L} and RBM20^{R634Q} mutations in human iPSCs and their orthologs in mouse models. While they could rescue the DCM phenotype by individually targeting each mutation, PE offers the advantage of providing one single molecular therapy for two genetically heterogeneous patient cohorts with either one of the two RBM20 mutations. Due to the broad editing window, length of the RTT, and the accumulation of SNPs within a mutational hotspot region of RBM20, a single pegRNA can be designed to target several variants. For example, the RTT of RBM20-epegRNA-12 covers 10 different pathogenic SNPs located within codons 633–638 (P633L, R634W, R634L, R634Q, S635C, R636C, R636S, R636L, R636H, and P638S),[41] assuming a minimal right homology arm of the RTT of 5 bp is sufficient for nucleotide substitutions.[42]

We demonstrated this strategy in TA-HEK cells carrying both the RBM20^{P633L} and RBM20^{R634Q} mutation on the same allele, achieving efficient simultaneous correction. However, this strategy could not be transferred to hi-CMs, supporting the assumption of a synergistic effect of correcting the two RBM20 mutations simultaneously in TA-HEK cells. Li et al. showed that introducing a silent mutation close to the intended edit can enhance editing efficiencies, as the cell's MMR recognition is evaded.[43] In hi-CMs carrying either single-point mutation, this advantage is not provided and could alleviate PE of the R634Q mutation. Why this affects the R634Q but not the P6333L mutation and whether this caveat can be overcome by using an advanced Prime Editor version, e.g., a PE6 version or PE7,[44,45] should be tested further. Nevertheless, RBM20-epegRNA-12's ability to correct other pathogenic SNPs within the editing window of the RTT should be tested in the future to exploit the maximum potential of the PE strategy and to benefit genetically diverse patient cohorts through a single editing agent.

The final goal is to provide the RBM20^{P633L}-PE strategy to patients. The base editing approach by Grosch et al. can be taken as a functional benchmark for clinical application of the RBM20-PE strategy. Base editing of the RBM20^{P633L} mutation achieved approximately 30% correction *in vitro* and 18%–20% *in vivo*. These editing levels restored RBM20-dependent splicing to WT levels, improving ejection fraction and physiological defects in mice. Based on their work, we assume that our PE strategy has the potential to provide curative therapy in adults, as PE of the RBM20^{P633L} mutation reached similarly high editing levels *in vitro* (34% on average).

Nevertheless, the delivery of the editing components remains a main hurdle for the application of the PE strategy *in vivo*, including limitations of cargo size and pegRNA stability. Davis et al. have used a dual-AAV approach *in vivo*, reaching 11% of PE in the heart.[16] However, exclusive AAV-mediated delivery of the editing components poses high risks of genomic integration and increased off-target editing.[46]

Our data support a combination of transient strategies, which can be optimized further for *in vivo* delivery. We used chimeric AAV to express epegRNAs in hi-CMs reaching transduction rates with the AAV-DJ capsid of 80% (Figure S5). Based on prior work by Grosch et al., the unspecific D/J serotype should be exchanged for the cardiotropic capsid AAVMYO, which was shown to specifically target cardiomyocytes and skeletal muscle upon tail vein injection.[13] The transfection of PEmax- and MLH1dn-mRNA has proven to work efficiently in hi-CMs in our experiments and in previous work[47] and provides a foundation for cardiotropic mRNA-LNP-mediated delivery in future *in vivo* experiments.[48,49] Both delivery vehicles should be administered regionally via thoracic or intracoronary injections, as was performed by Nishiyama et al. with AAV9 in mice.[8] This hybrid delivery strategy minimizes editor exposure and fits within packaging constraints.

To prevent nuclease degradation and secondary structure assembly of the long pegRNA-3′ extension, a stabilizing motif is appended (epegRNAs). However, this motif renders the pegRNA architecture even more complex and requires careful optimization of delivery modalities. An et al. have tackled this challenge by optimizing virus-like particle (VLP)-mediated delivery of PE and epegRNAs.[50] VLPs package PEmax and epegRNAs as ribonucleoprotein particles (RNPs), offering a fully transient, viral-free delivery that we consider promising for future *in vivo* application of the RBM20-strategy.

We focused on the PEmax version in our study, which was adequate for our intention to establish PE in hi-CMs. Now, more optimized PE variants with higher editing efficiencies have been developed,[44,45,51] but their potential to improve the performance of PE in hi-CMs remains to be determined. Our platform can be readily adapted to evaluate these and future PE versions.

In summary, we established a modular system for pegRNA screening in accessible HEK293T cells (TA-HEK) followed by functional validation in post-mitotic human hi-CMs. Our results demonstrate for the first time that PE can efficiently correct clinically relevant DCM-causing mutations to WT and rescue downstream molecular defects in a human post-mitotic model. At the same time, our study

highlights that editing efficiency is highly mutation- and context-dependent. These findings pave the way for optimizing *in vivo* strategies to treat RBM20^{P633L}-mediated DCM and other inherited cardiac diseases.

MATERIALS AND METHODS

Plasmid generation

For adenine base editing in Venus-reporter HEK cells, pCMV-ABE7.10 (Addgene #102919) was cotransfected with an sgRNA expressed from a pU6 plasmid. PE2- and PEmax-p2A-GFP plasmids were acquired from Addgene (#132776 and #180020). pegRNAs were cloned into the pU6-GG-acceptor plasmid (Addgene #132777) with Golden Gate assembly according to Doman et al.[33] pegRNA sequences are listed in Table S1. epegRNAs were cloned into the pU6-tmpknot-GG-acceptor (Addgene #174039) by amplifying the pegRNAs from the pU6-pegRNA-GG-acceptor plasmid.

The entire epegRNA cassette was subcloned into a pAAV-backbone by amplifying the epegRNAs with primers binding to the U6 promoter and mpknot, respectively (Table S2). The respective nicking sgRNA (Table S3) was cloned into the pAAV-epegRNA plasmid by amplifying the sgRNA from an sgRNA-expressing pU6 plasmid (Table S2). Correct plasmid assembly was confirmed by whole plasmid sequencing (PlasmidSaurus).

rAAV production and transduction

rAAV-D/J was produced as previously described.[52] Briefly, HEK293T cells were plated on 150-mm dishes and transfected with 10-μg pAAV-helper, 5-μg pRep/Cap-DJ, and 5-μg transgene plasmid per dish using a PEI:DNA ratio at 3:1. Five days later, rAAV was precipitated from the medium using polyethylene glycol (PEG) and extracted from the cells by four rounds of freeze-thawing. Residual DNA plasmids and non-encapsulated viral DNA were removed by benzonase treatment (50 U/mL, VWR International). The solution was loaded over an iodixanol gradient (Sigma-Aldrich) of four phases (15%, 25%, 40%, and 60%) and ultracentrifuged at 200,000×g for 2 h at 18°C in a Beckman Type 70Ti rotor. The rAAV-containing phase was obtained by puncturing the centrifugation tube (Beckman Coulter) at the height of the 40%/60 % interface with an 18-g needle. The rAAV-containing iodixanol solution was filtered through a 0.22-μm PES syringe filter (Thermo Fisher Scientific) and dialyzed to exchange the buffer to PBS and to increase the concentration. The purified rAAV solution was treated with DNase I (Qiagen) to remove residual DNA plasmids. RT-qPCR with a TaqMan probe and primers binding to the inverted terminal repeats (ITRs) was performed to quantify the rAAV titer. Hi-CMs were transduced with 1×10^6 vg/cell on day 21 of differentiation. The rAAV was removed from the cells 2 days after transduction.

In vitro transcription of PEmax- and MLH1dn-mRNA

In vitro transcription (IVT) of PEmax-/MLH1dn-mRNA was performed as previously published by Doman et al.[33] The linear IVT template was amplified from the pT7-PEmax (Addgene #178113) and pT7-hMLH1dn (Addgene #178114) plasmids with a GXL polymerase (Takara Bio), a forward primer correcting the point mutation within the T7 promoter, and a reverse primer appending a 120-bp-long polyA tail to the 3′ UTR (Table S2). The PCR product was verified on a 1% agarose gel and purified with a GeneJET PCR Purification Kit (Thermo Fisher Scientific). One microgram of purified template was used for IVT with the HiScribe T7 High Yield RNA Synthesis Kit (NEB) with N1-methyl-pseudouridine (TriLink Biotechnologies) instead of uridine and co-transcriptional capping with CleanCap AG (TriLink Biotechnologies). Template DNA was removed with DNase I (Qiagen). The synthesized RNA was purified using the RNA Cleanup Kit (Zymo Research) and eluted in RNase-free water. IVT samples and Millennium ssRNA Ladder (Thermo Fisher Scientific) were loaded to a 1.5% agarose gel to verify correct band size and purity. mRNA concentration was determined using a NanoDrop and stored as aliquots at −70°C until usage.

Generation of stable transgenic target-array-HEK293T cell line

The TA consists of a 156-bp-long fragment of the LMNA gene (including the K117fs mutation) and a 174 bp stretch of the RBM20 locus (including both the P633L and R634Q mutations) with a PacI restriction site at the 5′-end and an AsiSI restriction site at the 3′-end. The 348-bp-long TA was synthesized as a g-Block (IDT) and first cloned into a pJet vector using the CloneJET PCR Cloning Kit (Thermo Fisher Scientific). Correct ligation was verified by Sanger sequencing (LGC Genomic) using a primer binding to the pJet backbone (Table S2). For delivery of the TA into HEK293T cells, the TA was subcloned into a backbone containing a puromycin selection marker flanked by AAVS1 homology arms. To this purpose, the insert and backbone were digested with PacI and AsiSI restriction enzymes and ligated using T4 ligase. Correct integration was confirmed by Sanger sequencing with a primer binding to the backbone (Table S2). For stable integration of the Puro-TA in the AAVS1 locus of HEK293T cells, WT HEK293T cells were cotransfected with 3 μg of the Puro-TA-donor plasmid and 3 μg of a single plasmid expressing the AAVS1-sgRNA (Table S3) from a U6 promoter and SpCas9 and a Venus reporter under the control of a CAG promoter using PEI. After 2 days of transfection, the cells were supplied daily with fresh medium containing 1μg/mL puromycin (Thermo Fisher Scientific) for selection of TA-positive cells. After 8 days, Venus-positive cells were FACS-sorted, and single clones were replated into a well of a 96-well plate. Single cells were cultured in normal medium until colonies were formed. Correct integration of the Puro-TA was confirmed by GXL-PCR (Takara Bio) amplification on gDNA from single colonies using primers binding to the AAVS1 locus (Table S2).

Cell culture

WT HEK293T, Venus-reporter HEK, and target-array HEK cells were cultured in DMEM+GlutaMAX (Thermo Fisher Scientific) supplemented with 10% (v/V) fetal bovine serum (FBS) and 5% (v/V) Pen/Strep (Thermo Fisher Scientific). Cells were cultured at 37°C with 5% CO_2.

Culture conditions for human iPSCs and cardiac differentiation

Patient-derived, isogenic WT and mutant iPSCs were previously generated and characterized.[20–22] iPSCs were obtained from the MDC Technology Platform Pluripotent Stem Cells and the EMBL Heidelberg.

iPSCs were cultured on Geltrex (Thermo Fisher Scientific)-coated plates in Essential 8 Flex (E8, STEMCELL Technologies) medium and passaged with 0.5 mM EDTA/PBS. At 90% confluence, cardiac differentiation was initiated by changing the medium to RPMI-1640 (Thermo Fisher Scientific) supplemented with CHIR-99021 (6–9 μM, Selleckchem) and B−27 without insulin (Thermo Fisher Scientific).[38] After 24 h, CHIR was diluted by adding an equal volume of RPMI supplemented with B27-Insulin to the cells. At day 3, the medium was replaced with RPMI-Insulin and 5 μM IWR-1-endo (Selleckchem). From day 7 onward, cells were cultured in RPMI with complete B27 supplement (maintenance medium, Thermo Fisher Scientific), which was changed every 2–3 days. Cardiomyocytes (hi-CMs) were purified by metabolic selection in RPMI-1640 without glucose (Thermo Fisher Scientific), supplemented with sodium DL-lactate (Sigma-Aldrich) and chemically defined medium supplement (CDM3) for 3 days (days 12–15). hi-CMs were dissociated, frozen, and thawed as previously published.[53] Briefly, hi-CMs were replated to 12-well plates and 8-well μ-slides (Ibidi) using 10× TrypLE (Thermo Fisher Scientific) and maintenance medium supplemented with 10% knockout serum replacement (KOSR, Thermo Fisher Scientific) and RevitaCell supplement (Thermo Fisher Scientific) between days 16 and 19. The medium was changed back to maintenance medium the following day. rAAVs were added to the hi-CMs at day 21. hi-CMs were transfected with PEmax-/MLH1dn-mRNA the following day without removing the rAAVs and incubated for 18 h. Cells were harvested for genomic analysis on day 27. RNA and protein analysis was performed on day 34 of differentiation.

Transfection in HEK293T cells and hi-CMs

WT, Venus reporter, and TA-HEK cells were cotransfected with a plasmid encoding base or prime editor (1 μg) and a plasmid delivering the sgRNA, pegRNA, epegRNA, or nicking sgRNA (1 μg) using PEI in a 1:3 ratio. The medium was exchanged 18 h after transfection; 5×10^5 hi-CMs were transfected with 2 μg PEmax-mRNA and 0.5 μg MLH1dn-mRNA on a 24-well plate using 7.5 μL LipoStem transfection reagent (1:3 ratio of mRNA to LipoStem, Thermo Fisher Scientific) according to the manufacturer's instructions. The transfection reagent was removed 18 h after transfection.

Flow cytometry

Venus- and GFP-expressing HEK cells were quantified and sorted using a BD Aria III flow cytometer (BD Biosciences). In brief, Venus-reporter HEK cells were harvested 18 days and TA-HEK cells 3 days post-transfection. At least 10,000 viable single cells were recorded per sample for quantification; 300,000 cells expressing the respective reporter were sorted for genomic DNA extraction. Sorted cells were collected in DMEM+GlutaMAX. Data were analyzed with the FlowJo software (Tree Star; v.10.8.1).

Genome sequencing and data analysis

gDNA was isolated from TA-HEKs and hi-CMs using the Quick-DNA Miniprep Kit (Zymo Research) and eluted in ddH20. PCR amplification was performed with Q5 High-Fidelity DNA Polymerase (NEB) for on-target analysis from TA-HEK cells and LMNA-hi-CMs. The RBM20 locus was PCR-amplified with LongAmp Taq DNA Polymerase (NEB) for Sanger sequencing and with GXL polymerase (Takara Bio) for amplicon sequencing. Respective primer sequences are provided in Table S2. Sanger sequencing was quantified using the web tool DECODR v.3.0.[54] Next-generation amplicon sequencing was performed at GENEWIZ (Amplicon EZ service) using an Illumina MiSeq platform and 250 bp paired-end reads. Results were analyzed using CRISPResso2.[55]

RNA isolation and RT-PCR

Total RNA was isolated from cultured hi-CMs using TRIzol (Thermo Fisher Scientific) following the manufacturer's protocol. cDNA was generated from 2 μg total RNA using the High-Capacity cDNA Reverse Transcription Kit (Thermo Fisher Scientific). CAMK2D splice PCR was performed using Q5 High-Fidelity DNA polymerase (NEB) with primers listed in Table S2.[56] Densitometry analysis of the agarose gel was performed with ImageJ (v.2.16.0).

Immunocytochemistry of iPSC-CMs

hi-CMs grown on IbiTreat-coated 8-well μ-slides (Ibidi) were fixed with 4% paraformaldehyde (PFA) for 15 min. Fixed cells were permeabilized with 0.05% Tween 20 (Sigma-Aldrich) and 0.025% Triton X-100 (Carl Roth) and simultaneously blocked with 1% bovine serum albumin (BSA; Sigma-Aldrich) for 1 hour at room temperature. Primary antibodies were incubated overnight at 4°C before incubating DAPI and Alexa-Fluor-conjugated secondary antibodies at room temperature for 45 min. Primary antibodies include rabbit anti-RBM20 (Cat. No.: NBP2-34038, Novus Biologicals, 1:250), mouse anti-Actinin (Cat. No.: A7811, Sigma-Aldrich, 1:400), and rabbit anti-Ki67 (Cat. No.: 9129, D3B5, Cell signaling, 1:400). Secondary antibodies include goat anti-rabbit Alexa Fluor 555 and goat anti-mouse Alexa 488. Images were acquired with a confocal TCS SP8 DLS microscope (Leica Microsystems) using a 63× glycerol objective.

DAPI:RBM20 spatial correlation analysis

Correlation of RBM20 and DAPI was analyzed using the ImageJ (v.2.16.0) plugin Coloc2 according to Kornienko et al.[39] A region of interest (ROI) was created covering at least five nuclei, which was used as ROI/mask for determining the Pearson correlation coefficient R between the RBM20 and DAPI channel in single slices of a z-stack.

Statistics and reproducibility

Statistics and data analysis were performed with GraphPad Prism v.10.2.2. Data are shown as means with standard deviations (SDs).

Data distribution was assessed for normality using the Shapiro-Wilk test and Q-Q plot inspection. Sample sizes, *p* value, and the statistical tests used are described in the figure legends. A *p* value of less than 0.05 was considered statistically significant. No statistical method was used to predetermine sample size. No data were excluded from the analyses. The experiments were not randomized, and the investigators were not blinded to allocation during experiments and outcome assessment.

DATA AND CODE AVAILABILITY

- The main data are available in the main text or the supplemental information.
- Illumina sequencing data are available in the Sequence Read Archive under the BioProject accession number: PRJNA1285140.
- Additional data and/or additional files are available via corresponding authors upon reasonable request.

ACKNOWLEDGMENTS

We thank the Max-Delbrück Center Berlin-Buch flow cytometry technology platform and the Advanced Light Microscopy technology platform for technical support and access to instruments. We are grateful to the Technology Platform Pluripotent Stem Cells for providing LMNA-iPSC lines, for their support during the establishment of cardiac differentiation protocols and assistance with cardiac differentiations.

AUTHOR CONTRIBUTIONS

T.D. and R.K. provided the conceptual framework for the study and administered the projects. A.R., T.D., and R.K. designed the study. M.G. and L.M.S. provided the RBM20-iPSC lines. A.R. and A.Z. performed the experiments. A.R. analyzed the data and wrote the original manuscript. T.D. and R.K. participated in the review and editing of the manuscript.

DECLARATION OF INTERESTS

The authors declare no competing interests.

DECLARATION OF GENERATIVE AI AND AI-ASSISTED TECHNOLOGIES IN THE WRITING PROCESS

During the preparation of this work, the authors used ChatGPT4 in order to refine sentence structure. After using this tool, the authors reviewed and edited the content as needed and take full responsibility for the content of the publication.

SUPPLEMENTAL INFORMATION

Supplemental information can be found online at https://doi.org/10.1016/j.omtn.2025.102734.

REFERENCES

1. Anzalone, A.V., Randolph, P.B., Davis, J.R., Sousa, A.A., Koblan, L.W., Levy, J.M., Chen, P.J., Wilson, C., Newby, G.A., Raguram, A., and Liu, D.R. (2019). Search-and-replace genome editing without double-strand breaks or donor DNA. Nature *576*, 149–157. https://doi.org/10.1038/s41586-019-1711-4.

2. McNally, E.M., and Mestroni, L. (2017). Dilated Cardiomyopathy. Circ. Res. *121*, 731–748. https://doi.org/10.1161/CIRCRESAHA.116.309396.

3. Rosenbaum, A.N., Agre, K.E., and Pereira, N.L. (2020). Genetics of dilated cardiomyopathy: practical implications for heart failure management. Nat. Rev. Cardiol. *17*, 286–297. https://doi.org/10.1038/s41569-019-0284-0.

4. McGurk, K.A., Zhang, X., Theotokis, P., Thomson, K., Harper, A., Buchan, R.J., Mazaika, E., Ormondroyd, E., Wright, W.T., Macaya, D., et al. (2023). The penetrance of rare variants in cardiomyopathy-associated genes: A cross-sectional approach to estimating penetrance for secondary findings. Am. J. Hum. Genet. *110*, 1482–1495. https://doi.org/10.1016/j.ajhg.2023.08.003.

5. Hershberger, R.E., Hedges, D.J., and Morales, A. (2013). Dilated cardiomyopathy: the complexity of a diverse genetic architecture. Nat. Rev. Cardiol. *10*, 531–547. https://doi.org/10.1038/nrcardio.2013.105.

6. Parks, S.B., Kushner, J.D., Nauman, D., Burgess, D., Ludwigsen, S., Peterson, A., Li, D., Jakobs, P., Litt, M., Porter, C.B., et al. (2008). Lamin A/C mutation analysis in a cohort of 324 unrelated patients with idiopathic or familial dilated cardiomyopathy. Am. Heart J. *156*, 161–169. https://doi.org/10.1016/J.AHJ.2008.01.026.

7. Refaat, M.M., Lubitz, S.A., Makino, S., Islam, Z., Frangiskakis, J.M., Mehdi, H., Gutmann, R., Zhang, M.L., Bloom, H.L., MacRae, C.A., et al. (2012). Genetic variation in the alternative splicing regulator RBM20 is associated with dilated cardiomyopathy. Heart Rhythm *9*, 390–396. https://doi.org/10.1016/J.HRTHM.2011.10.016.

8. Nishiyama, T., Zhang, Y., Cui, M., Li, H., Sanchez-Ortiz, E., McAnally, J.R., Tan, W., Kim, J., Chen, K., Xu, L., et al. (2022). Precise genomic editing of pathogenic mutations in RBM20 rescues dilated cardiomyopathy. Sci. Transl. Med. *14*, eade1633. https://doi.org/10.1126/scitranslmed.ade1633.

9. Ma, S., Jiang, W., Liu, X., Lu, W.J., Qi, T., Wei, J., Wu, F., Chang, Y., Zhang, S., Song, Y., et al. (2021). Efficient Correction of a Hypertrophic Cardiomyopathy Mutation by ABEmax-NG. Circ. Res. *129*, 895–908. https://doi.org/10.1161/CIRCRESAHA.120.318674.

10. Chai, A.C., Cui, M., Chemello, F., Li, H., Chen, K., Tan, W., Atmanli, A., McAnally, J. R., Zhang, Y., Xu, L., et al. (2023). Base editing correction of hypertrophic cardiomyopathy in human cardiomyocytes and humanized mice. Nat. Med. *29*, 401–411. https://doi.org/10.1038/s41591-022-02176-5.

11. Yang, L., Liu, Z., Sun, J., Chen, Z., Gao, F., and Guo, Y. (2024). Adenine base editor-based correction of the cardiac pathogenic LMNA c.1621C > T mutation in murine hearts. J. Cell Mol. Med. *28*, e18145. https://doi.org/10.1111/JCMM.18145.

12. Reichart, D., Newby, G.A., Wakimoto, H., Lun, M., Gorham, J.M., Curran, J.J., Raguram, A., DeLaughter, D.M., Conner, D.A., Marsiglia, J.D.C., et al. (2023). Efficient *in vivo* genome editing prevents hypertrophic cardiomyopathy in mice. Nat. Med. *29*, 412–421. https://doi.org/10.1038/s41591-022-02190-7.

13. Grosch, M., Schraft, L., Chan, A., Küchenhoff, L., Rapti, K., Ferreira, A.M., Kornienko, J., Li, S., Radke, M.H., Krämer, C., et al. (2023). Striated muscle-specific base editing enables correction of mutations causing dilated cardiomyopathy. Nat. Commun. *14*, 3714–3715. https://doi.org/10.1038/s41467-023-39352-1.

14. "Cardiac Disorder" - ClinVar - NCBI. https://www.ncbi.nlm.nih.gov/clinvar.

15. Yao, B., Lei, Z., Gonçalves, M.A.F.V., and Sluijter, J.P.G. (2024). Integrating Prime Editing and Cellular Reprogramming as Novel Strategies for Genetic Cardiac Disease Modeling and Treatment. Curr. Cardiol. Rep. *26*, 1197–1208. https://doi.org/10.1007/s11886-024-02118-2.

16. Davis, J.R., Banskota, S., Levy, J.M., Newby, G.A., Wang, X., Anzalone, A.V., Nelson, A.T., Chen, P.J., Hennes, A.D., An, M., et al. (2024). Efficient prime editing in mouse brain, liver and heart with dual AAVs. Nat. Biotechnol. *42*, 253–264. https://doi.org/10.1038/s41587-023-01758-z.

17. Chemello, F., Chai, A.C., Li, H., Rodriguez-Caycedo, C., Sanchez-Ortiz, E., Atmanli, A., Mireault, A.A., Liu, N., Bassel-Duby, R., and Olson, E.N. (2021). Precise correction of Duchenne muscular dystrophy exon deletion mutations by base and prime editing. Sci. Adv. *7*, eabg4910. https://doi.org/10.1126/sciadv.abg4910.

18. Wang, Q., Capelletti, S., Liu, J., Janssen, J.M., and Gonçalves, M.A.F.V. (2024). Selection-free precise gene repair using high-capacity adenovector delivery of advanced prime editing systems rescues dystrophin synthesis in DMD muscle cells. Nucleic Acids Res. *52*, 2740–2757. https://doi.org/10.1093/NAR/GKAE057.

19. Chen, P.J., Hussmann, J.A., Yan, J., Knipping, F., Ravisankar, P., Chen, P.F., Chen, C., Nelson, J.W., Newby, G.A., Sahin, M., et al. (2021). Enhanced prime editing systems by manipulating cellular determinants of editing outcomes. Cell *184*, 5635–5652.e29. https://doi.org/10.1016/j.cell.2021.09.018.

20. Brauch, K.M., Karst, M.L., Herron, K.J., de Andrade, M., Pellikka, P.A., Rodeheffer, R.J., Michels, V.V., and Olson, T.M. (2009). Mutations in Ribonucleic Acid Binding Protein Gene Cause Familial Dilated Cardiomyopathy. J. Am. Coll. Cardiol. *54*, 930–941. https://doi.org/10.1016/j.jacc.2009.05.038.

21. Lee, J., Termglinchan, V., Diecke, S., Itzhaki, I., Lam, C.K., Garg, P., Lau, E., Greenhaw, M., Seeger, T., Wu, H., et al. (2019). Activation of PDGF pathway links LMNA mutation to dilated cardiomyopathy. Nature *572*, 335–340. https://doi.org/10.1038/s41586-019-1406-x.

22. Briganti, F., Sun, H., Wei, W., Wu, J., Zhu, C., Liss, M., Karakikes, I., Rego, S., Cipriano, A., Snyder, M., et al. (2020). iPSC Modeling of RBM20-Deficient DCM Identifies Upregulation of RBM20 as a Therapeutic Strategy. Cell Rep. *32*, 108117–108121. https://doi.org/10.1016/j.celrep.2020.108117.

23. Guo, W., Schafer, S., Greaser, M.L., Radke, M.H., Liss, M., Govindarajan, T., Maatz, H., Schulz, H., Li, S., Parrish, A.M., et al. (2012). RBM20, a gene for hereditary cardiomyopathy, regulates titin splicing. Nat. Med. *18*, 766–773. https://doi.org/10.1038/nm.2693.

24. Murayama, R., Kimura-Asami, M., Togo-Ohno, M., Yamasaki-Kato, Y., Naruse, T. K., Yamamoto, T., Hayashi, T., Ai, T., Spoonamore, K.G., Kovacs, R.J., et al. (2018). Phosphorylation of the RSRSP stretch is critical for splicing regulation by RNA-Binding Motif Protein 20 (RBM20) through nuclear localization. Sci. Rep. *8*, 8970. https://doi.org/10.1038/S41598-018-26624-W.

25. Schneider, J.W., Oommen, S., Qureshi, M.Y., Goetsch, S.C., Pease, D.R., Sundsbak, R.S., Guo, W., Sun, M., Sun, H., Kuroyanagi, H., et al. (2020). Dysregulated ribonucleoprotein granules promote cardiomyopathy in RBM20 gene-edited pigs. Nat. Med. *26*, 1788–1800. https://doi.org/10.1038/s41591-020-1087-x.

26. Fenix, A.M., Miyaoka, Y., Bertero, A., Blue, S.M., Spindler, M.J., Tan, K.K.B., Perez-Bermejo, J.A., Chan, A.H., Mayerl, S.J., Nguyen, T.D., et al. (2021). Gain-of-function cardiomyopathic mutations in RBM20 rewire splicing regulation and re-distribute ribonucleoprotein granules within processing bodies. Nat. Commun. *12*, 6324. https://doi.org/10.1038/s41467-021-26623-y.

27. Maatz, H., Jens, M., Liss, M., Schafer, S., Heinig, M., Kirchner, M., Adami, E., Rintisch, C., Dauksaite, V., Radke, M.H., et al. (2014). RNA-binding protein RBM20 represses splicing to orchestrate cardiac pre-mRNA processing. J. Clin. Investig. *124*, 3419–3430. https://doi.org/10.1172/JCI74523.

28. Wyles, S.P., Li, X., Hrstka, S.C., Reyes, S., Oommen, S., Beraldi, R., Edwards, J., Terzic, A., Olson, T.M., and Nelson, T.J. (2016). Modeling structural and functional deficiencies of RBM20 familial dilated cardiomyopathy using human induced pluripotent stem cells. Hum. Mol. Genet. *25*, 254–265. https://doi.org/10.1093/hmg/ddv468.

29. Hsu, J.Y., Grünewald, J., Szalay, R., Shih, J., Anzalone, A.V., Lam, K.C., Shen, M.W., Petri, K., Liu, D.R., Joung, J.K., and Pinello, L. (2021). PrimeDesign software for rapid and simplified design of prime editing guide RNAs. Nat. Commun. *12*, 1034. https://doi.org/10.1038/s41467-021-21337-7.

30. Chow, R.D., Chen, J.S., Shen, J., and Chen, S. (2021). A web tool for the design of prime-editing guide RNAs. Nat. Biomed. Eng. *5*, 190–194. https://doi.org/10.1038/s41551-020-00622-8.

31. Anderson, M.V., Haldrup, J., Thomsen, E.A., Wolff, J.H., and Mikkelsen, J.G. (2021). pegIT - a web-based design tool for prime editing. Nucleic Acids Res. *49*, W505–W509. https://doi.org/10.1093/NAR/GKAB427.

32. Mathis, N., Allam, A., Kissling, L., Marquart, K.F., Schmidheini, L., Solari, C., Balázs, Z., Krauthammer, M., and Schwank, G. (2023). Predicting prime editing efficiency and product purity by deep learning. Nat. Biotechnol. *41*, 1151–1159. https://doi.org/10.1038/s41587-022-01613-7.

33. Doman, J.L., Sousa, A.A., Randolph, P.B., Chen, P.J., and Liu, D.R. (2022). Designing and executing prime editing experiments in mammalian cells. Nat. Protoc. *17*, 2431–2468. https://doi.org/10.1038/s41596-022-00724-4.

34. Koeppel, J., Weller, J., Peets, E.M., Pallaseni, A., Kuzmin, I., Raudvere, U., Peterson, H., Liberante, F.G., and Parts, L. (2023). Prediction of prime editing insertion efficiencies using sequence features and DNA repair determinants. Nat. Biotechnol. *41*, 1446–1456. https://doi.org/10.1038/s41587-023-01678-y.

35. Kim, H.K., Yu, G., Park, J., Min, S., Lee, S., Yoon, S., and Kim, H.H. (2021). Predicting the efficiency of prime editing guide RNAs in human cells. Nat. Biotechnol. *39*, 198–206. https://doi.org/10.1038/s41587-020-0677-y.

36. Nelson, J.W., Randolph, P.B., Shen, S.P., Everette, K.A., Chen, P.J., Anzalone, A.V., An, M., Newby, G.A., Chen, J.C., Hsu, A., and Liu, D.R. (2022). Engineered pegRNAs improve prime editing efficiency. Nat. Biotechnol. *40*, 402–410. https://doi.org/10.1038/s41587-021-01039-7.

37. Choudhury, S., Huang, A.Y., Kim, J., Zhou, Z., Morillo, K., Maury, E.A., Tsai, J.W., Miller, M.B., Lodato, M.A., Araten, S., et al. (2022). Somatic mutations in single human cardiomyocytes reveal age-associated DNA damage and widespread oxidative genotoxicity. Nat. Aging *2*, 714–725. https://doi.org/10.1038/s43587-022-00261-5.

38. Burridge, P.W., Matsa, E., Shukla, P., Lin, Z.C., Churko, J.M., Ebert, A.D., Lan, F., Diecke, S., Huber, B., Mordwinkin, N.M., et al. (2014). Chemically defined generation of human cardiomyocytes. Nat. Methods *11*, 855–860. https://doi.org/10.1038/nmeth.2999.

39. Kornienko, J., Rodríguez-Martínez, M., Fenzl, K., Hinze, F., Schraivogel, D., Grosch, M., Tunaj, B., Lindenhofer, D., Schraft, L., Kueblbeck, M., et al. (2023). Mislocalization of pathogenic RBM20 variants in dilated cardiomyopathy is caused by loss-of-interaction with Transportin-3. Nat. Commun. *14*, 4312. https://doi.org/10.1038/s41467-023-39965-6.

40. Sousa, A.A., Hemez, C., Lei, L., Traore, S., Kulhankova, K., Newby, G.A., Doman, J. L., Oye, K., Pandey, S., Karp, P.H., et al. (2025). Systematic optimization of prime editing for the efficient functional correction of CFTR F508del in human airway epithelial cells. Nat. Biomed. Eng. *9*, 7–21. https://doi.org/10.1038/s41551-024-01233-3.

41. "RBM20"[GENE] - ClinVar - NCBI. https://www.ncbi.nlm.nih.gov/clinvar.

42. Yu, G., Kim, H.K., Park, J., Kwak, H., Cheong, Y., Kim, D., Kim, J., Kim, J., and Kim, H.H. (2023). Prediction of efficiencies for diverse prime editing systems in multiple cell types. Cell *186*, 2256–2272.e23. https://doi.org/10.1016/j.cell.2023.03.034.

43. Li, X., Zhou, L., Gao, B.-Q., Li, G., Wang, X., Wang, Y., Wei, J., Han, W., Wang, Z., Li, J., et al. (2022). Highly efficient prime editing by introducing same-sense mutations in pegRNA or stabilizing its structure. Nat. Commun. *13*, 1669. https://doi.org/10.1038/s41467-022-29339-9.

44. Doman, J.L., Pandey, S., Neugebauer, M.E., An, M., Davis, J.R., Randolph, P.B., McElroy, A., Gao, X.D., Raguram, A., Richter, M.F., et al. (2023). Phage-assisted evolution and protein engineering yield compact, efficient prime editors. Cell *186*, 3983–4002.e26. https://doi.org/10.1016/j.cell.2023.07.039.

45. Yan, J., Oyler-Castrillo, P., Ravisankar, P., Ward, C.C., Levesque, S., Jing, Y., Simpson, D., Zhao, A., Li, H., Yan, W., et al. (2024). Improving prime editing with an endogenous small RNA-binding protein. Nature *628*, 639–647.

46. Greig, J.A., Martins, K.M., Breton, C., Lamontagne, R.J., Zhu, Y., He, Z., White, J., Zhu, J.X., Chichester, J.A., Zheng, Q., et al. (2024). Integrated vector genomes may contribute to long-term expression in primate liver after AAV administration. Nat. Biotechnol. *42*, 1232–1242. https://doi.org/10.1038/s41587-023-01974-7.

47. Tan, S., Tao, Z., Loo, S., Su, L., Chen, X., and Ye, L. (2019). Non-viral vector based gene transfection with human induced pluripotent stem cells derived cardiomyocytes. Sci. Rep. *9*, 14404. https://doi.org/10.1038/s41598-019-50980-w.

48. Herrera-Barrera, M., Gautam, M., Lokras, A., Vlasova, K., Foged, C., and Sahay, G. (2023). Lipid Nanoparticle-Enabled Intracellular Delivery of Prime Editors. AAPS J. *25*, 65. https://doi.org/10.1208/s12248-023-00833-2.

49. Hołubowicz, R., Du, S.W., Felgner, J., Smidak, R., Choi, E.H., Palczewska, G., Menezes, C.R., Dong, Z., Gao, F., Medani, O., et al. (2024). Safer and efficient base editing and prime editing via ribonucleoproteins delivered through optimized lipid-nanoparticle formulations. Nat. Biomed. Eng. *9*, 57–78. https://doi.org/10.1038/S41551-024-01296-2.

50. An, M., Raguram, A., Du, S.W., Banskota, S., Davis, J.R., Newby, G.A., Chen, P.Z., Palczewski, K., and Liu, D.R. (2024). Engineered virus-like particles for transient delivery of prime editor ribonucleoprotein complexes *in vivo*. Nat. Biotechnol. *42*, 1526–1537. https://doi.org/10.1038/s41587-023-02078-y.

51. Weber, Y., Böck, D., Ivaşcu, A., Mathis, N., Rothgangl, T., Ioannidi, E.I., Blaudt, A. C., Tidecks, L., Vadovics, M., Muramatsu, H., et al. (2024). Enhancing prime editor activity by directed protein evolution in yeast. Nat. Commun. *15*, 1–11. https://doi.org/10.1038/s41467-024-46107-z.

52. Tran, N.T., Trombke, J., Rajewsky, K., and Chu, V.T. (2020). Protocol for efficient CRISPR/Cas9/AAV-mediated homologous recombination in mouse hematopoietic stem and progenitor cells. STAR Protoc. *1*, 100028. https://doi.org/10.1016/j.xpro.2020.100028.

53. Miller, D.C., Genehr, C., Telugu, N.S., Kurths, S., and Diecke, S. (2020). Simple Workflow and Comparison of Media for hPSC-Cardiomyocyte Cryopreservation and Recovery. Curr. Protoc. Stem Cell Biol. *55*, e125. https://doi.org/10.1002/cpsc.125.

54. Bloh, K., Kanchana, R., Bialk, P., Banas, K., Zhang, Z., Yoo, B.C., and Kmiec, E.B. (2021). Deconvolution of Complex DNA Repair (DECODR): Establishing a Novel Deconvolution Algorithm for Comprehensive Analysis of CRISPR-Edited Sanger Sequencing Data. CRISPR J. *4*, 120–131. https://doi.org/10.1089/crispr.2020.0022.

55. Clement, K., Rees, H., Canver, M.C., Gehrke, J.M., Farouni, R., Hsu, J.Y., Cole, M.A., Liu, D.R., Joung, J.K., Bauer, D.E., and Pinello, L. (2019). CRISPResso2 provides accurate and rapid genome editing sequence analysis. Nat. Biotechnol. *37*, 224–226. https://doi.org/10.1038/s41587-019-0032-3.

56. Radke, M.H., Badillo-Lisakowski, V., Britto-Borges, T., Kubli, D.A., Jüttner, R., Parakkat, P., Carballo, J.L., Hüttemeister, J., Liss, M., Hansen, A., et al. (2021). Therapeutic inhibition of RBM20 improves diastolic function in a murine heart failure model and human engineered heart tissue. Sci. Transl. Med. *13*, eabe8952. https://doi.org/10.1126/scitranslmed.abe8952.

OMTN, Volume *36*

Supplemental information

Prime editing corrects the dilated cardiomyopathy causing RBM20-P633L-mutation in human cardiomyocytes

Alexandra Roman, Anja Zimmer, Michael Gotthardt, Lars M. Steinmetz, Ralf Kühn, and Tu Dang

Supplemental Tables

Table S1 Sequences of pegRNAs

pegRNA#	Spacer		Extension		
	#	sequence (5’>3’)	RT [bp]	PBS [bp]	sequence (5’>3’)
RBM20-01	1 (PAM disrupting)	CTCACAGATA<br>TGGCCCAGAA	14	14	GACCGCGGCCTTTCTGGGCCATATCT<br>GT
RBM20-02			26	13	ACCGGACTACGAGACcGCgGCCTTTCT<br>GGGCCATATCTG
RBM20-03			18	10	ACGAGACCGCGGtCTTTC<br>GGGCCATAT
RBM20-04			21	10	ACTACGAGACCGCGGTCTTTC<br>TGGGCCATAT
RBM20-05			24	10	CGGACTACGAGACCGCGGTCTTTCTG<br>GGCCATAT
RBM20-06			18	13	ACGAGACCGCGGTCTTTCTGGGCCAT<br>ATCTG
RBM20-07			21	13	ACTACGAGACCGCGGTCTTTCTGGGC<br>CATATCTG
RBM20-08			24	13	CGGACTACGAGACCGCGGTCTTTCTG<br>GGCCATATCTG
RBM20-09			18	15	ACGAGACCGCGGTCTTTCTGGGCCAT<br>ATCTGTG
RBM20-10			21	15	ACTACGAGACCGCGGTCTTTCTGGGC<br>CATATCTGTG
RBM20-11			24	15	CGGACTACGAGACCGCGGTCTTTCTG<br>GGCCATATCTGTG
RBM20-12	2	GGGGAGAGT<br>GACCGGCTCA<br>C	24	10	AGGCCGCGGTCTCGTAGTCCGGTGAG<br>CCGGTCAC
RBM20-13			22	12	GCCGCGGTCTCGTAGTCCGGTGAGCC<br>GGTCACTC
RBM20-14	3	CTTGGCTCCC<br>TCACAGATA	38	15	TCACCGGACTACGAGACcGCgGCCTTT<br>CTGGGCCATATCTGTGAGGGAGCCAA

RBM20-15			38	14	TCACCGGACTACGAGACcGCgGCCTTTCTGGGCCATATCTGTGAGGGAGCCA
RBM20-16			38	13	TCACCGGACTACGAGACcGCgGCCTTTCTGGGCCATATCTGTGAGGGAGCC
LMNA-01	1	CAAAGTGCGTGAGGAGTTTA	13	15	TTCAGCTCCTTAAACTCCTCACGCACTT
LMNA-02	2	GGAGCTGAGCAAAGTGCGTG	22	12	TTCAGCTCCTTAAACTCCTCACGCACTTTGCTCA
LMNA-03	3 (PAM disrupting)	GCGTGAGGAGTTTAAGGAGC	12	13	GCGCTTTCAGCTCCTTAAACTCCTC
LMNA-04			15	14	ACCGCGCTTTCAGCTCCTTAAACTCCTCA
LMNA-05			15	13	ACCGCGCTTTCAGCTCCTTAAACTCCTC
LMNA-07			13	10	CGCGCTTTCAGCTCCTTAAACTC
LMNA-08			16	10	CACCGCGCTTTCAGCTCCTTAAACTC
LMNA-09			19	10	ACTCACCGCGCTTTCAGCTCCTTAAACTC
LMNA-10			13	13	CGCGCTTTCAGCTCCTTAAACTCCTC
LMNA-11			16	13	CACCGCGCTTTCAGCTCCTTAAACTCCTC
LMNA-12			19	13	ACTCACCGCGCTTTCAGCTCCTTAAACTCCTC
LMNA-13			13	15	CGCGCTTTCAGCTCCTTAAACTCCTCAC
LMNA-14			16	15	CACCGCGCTTTCAGCTCCTTAAACTCCTCAC
LMNA-15			19	15	ACTCACCGCGCTTTCAGCTCCTTAAACTCCTCAC
LMNA-16			12	15	GCGCTTTcAGCTCCTTAAACTCCTCAC
LMNA-17			12	14	GCGCTTTcAGCTCCTTAAACTCCTCA
LMNA-18			15	15	ACCGCGCTTTcAGCTCCTTAAACTCCTCAC

Venus-01	1	GACGTAGCCT TCGGGCATGG	36	11	ACCACATGAAGcAGCACGACTTCTTCA AGTCCGCCATGCCCGAAGGC

Table S2 Primers

**Name**	**Sequence (5' > 3')**
IVT_PEmax_Fwd	TCGAGCTCGGTACCTAATACGACtCACTATAAGG
IVT_Rev	(T_{120})CTTCCTACTCAGGCTTTATTCAAAGACCA
epegRNA_Fwd	AATTCAGTCGACTGGATCCG
epegRNA_hRBM20-sg1-18RT-10PBS-Linker-rev	AGGGGGGGGGCTCCTGAATATGGCCCAGAAAG
epegRNA_hRBM20-sg2-24RT-10PBS-Linker-rev	AGGGGGGGGGCTCCTGAGTGACCGGCTCACCG
epegRNA_hRBM20-sg3-38RT-14PBS-Linker-rev	AGGGGGGGGGCTCCTGATGGCTCCCTCACAGA
epegRNA_LMNA_sg3-12RT-13PBS-Linker-Rev	AGGGGGGGGGCTCCTGAGAGGAGTTTAAGGAG
epegRNA_LMNA_sg3-19RT-10PBS-Linker-Rev	AGGGGGGGGGCTCCTGAGAGTTTAAGGAGCTG
epegRNA_LMNA_sg3-12RT-15PBS-Linker-Rev	AGGGGGGGGGCTCCTGAGTGAGGAGTTTAAGG
epegRNA_LMNA_sg3-12RT-14PBS-Linker-Rev	AGGGGGGGGGCTCCTGATGAGGAGTTTAAGGA
U6+AscI-Fw	CATGAGGCGCGCC GAGGGCCTATTTCCCATGATT
mpknot+AscI-Rev	AATCCGGCGCGCC AACGCCAGCAACGCGGCCTT
PE-sgRNA-AAV-HINDIII-Fw	AGGCAT AAGCTT GCAAGCGATCGCGGGGCCGC
PE-sgRNA-AAV-HINDIII-Rev	AGGCAT AAGCTT GGCGCGCCCACCCTTGGATC
pJet-Fwd	CGACTCACTATAGGGAGAGCGGC
pAAV-Puro-TA-Donor	GCTAACCATGTTCATGCCTTCTTC
AAVS1-Fw	CCCCTTACCTCTCTAGTCTGTGC
AAVS1-Rev	CTCAGGTTCTGGGAGAGGGTAG
hLMNA-geno-fw	CGAGTCTGAAGAGGTGGTCA
hLMNA-geno-rev	GGCAAATCCCAATCTGACCG
RBM20-geno-fw	AGGAGGAGAGTCAGAGGTCC
RBM20-geno-rev	TTTGGGCTCTTTCCGGTAGT
RBM20-geno-Illumina-fw	TGAGTAAAGGCACAGCGAGT
RBM20-geno-Illumina-rev	TGTCCCTCTTGTCATCTCCG
CAG2-fw	TTCGGCTTCTGGCGTGTGACC
bpA-rev	TAGAAGGCACAGTCGAGG
hCAMK2D_E12_fwd	AAGGGTGCCATCTTGACAAC

hCAMK2D_E16_rev	TGCTTTCGTGCTTTCACATC
PE_OT_01_fw	ACCTCTGCCCTCTGATCTGT
PE_OT_01_rev	AGGTCGCTTGCCTTCGTTTA
PE_OT_02_fw	CGGTGCTGTGACTGAGACAT
PE_OT_02_rev	ACAGCCCTGACTACTGTCCT
PE_OT_03_fw	GGAAGGACTCCCTGTCTCCT
PE_OT_03_rev	TCTGGAGAGAAGACTGGGCA
PE_OT_04_fw	GTTGACGGTAGCTGCTTCCT
PE_OT_04_rev	GCGTTTATGAGGGTGAGGCA
PE_OT_05_fw	TGACTGGGCTGCAGAGTTTT
PE_OT_05_rev	GCTGTAAACGTGTGATCGCC

Table S3 sgRNAs

Name	Sequence (5‘>3‘)
hRBM20-PE3b-ngRNA-1	TACGAGACCGCGGCCTTTCT
hRBM20-PE3b-ngRNA-2	GATATGGCCCAGAAAGGCCG
hRBM20-PE3b-ngRNA-3	TCACCGGACTACGAGACcG
hLMNA-PE3-ngRNA-1	CTGCAGGCGGGCGCGCTCCT
hLMNA-PE3-ngRNA-2	TACTGAGTCAAGGGTCTTGC
AAVS1-sgRNA	CTCAGGTTCTGGGAGAGGGTAG

Supplemental Figures

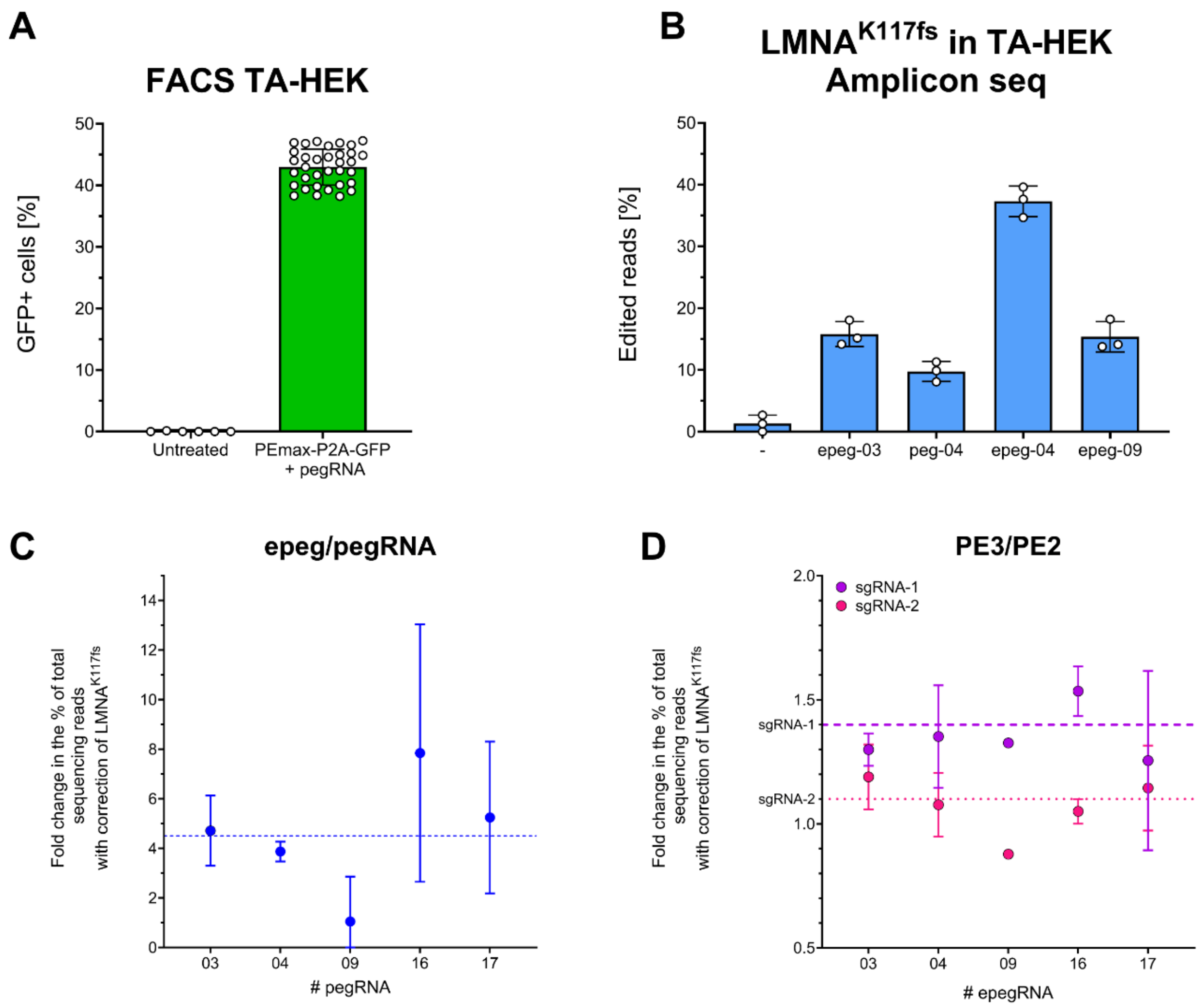

Figure S1: Profiling of pegRNA activity in TA-HEK cells. A Quantification of GFP+ TA-HEK-cells three days after co-transfection with PEmax-p2A-GFP-plasmid and a pegRNA analysed by FACS. Data are expressed as mean (SD; Untreated, n=6; PEmax-p2A-GFP+ pegRNA, n=34 independent experiments). **B** Amplicon sequencing of TA-HEK-cells treated with PE4 and one of five LMNA-(e)pegRNAs. All data are expressed as mean (SD; n=3 independent experiments). **C** Comparison of LMNA-editing efficiency when using epegRNA versus the corresponding pegRNA. Dotted line represents average

fold change across all epegRNAs tested. **D** Comparison of editing efficiency when using the PE2 (no nicking sgRNA) versus PE3 system with the nicking sgRNA-1 or -2. Dashed and dotted line represent average fold change across all LMNA-epegRNAs with sgRNA-1 or -2, respectively. C, D Each dot represents the mean value (SD) of n=3 independent experiments.

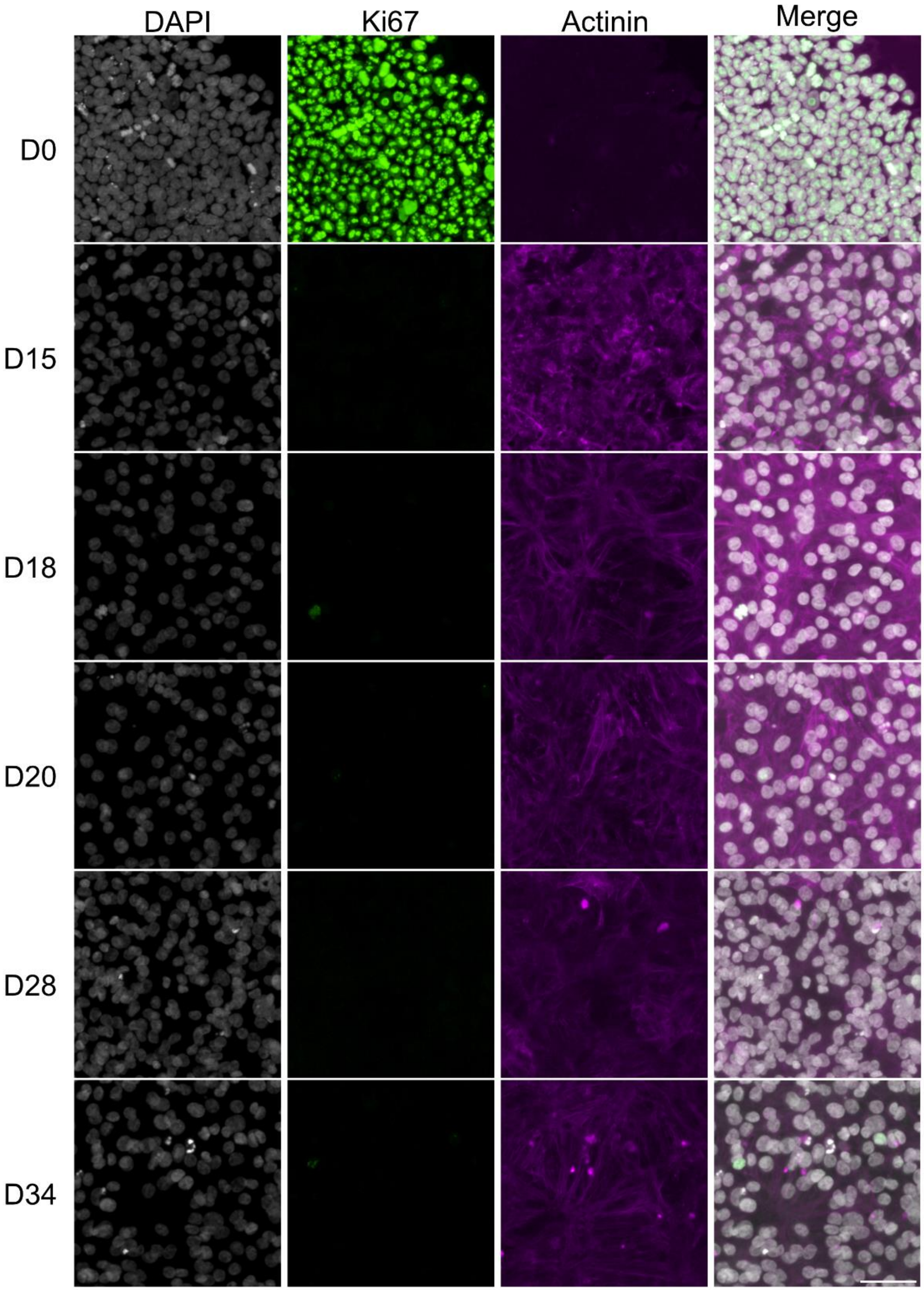
DAPI
Ki67
Actinin
Merge
D0
D15
D18
D20
D28
D34

Figure S2: Immunocytochemistry staining of proliferation and cardiac marker in hi-CMs. Representative images of homozygous RBM20^{P633L} iPSCs (day 0) and hi-CMs at day 15, 18, 20, 28 and 34. Cells were stained for DAPI (grey), Ki67 (green) and α-Actinin (magenta). Scale bar, 50 µm.

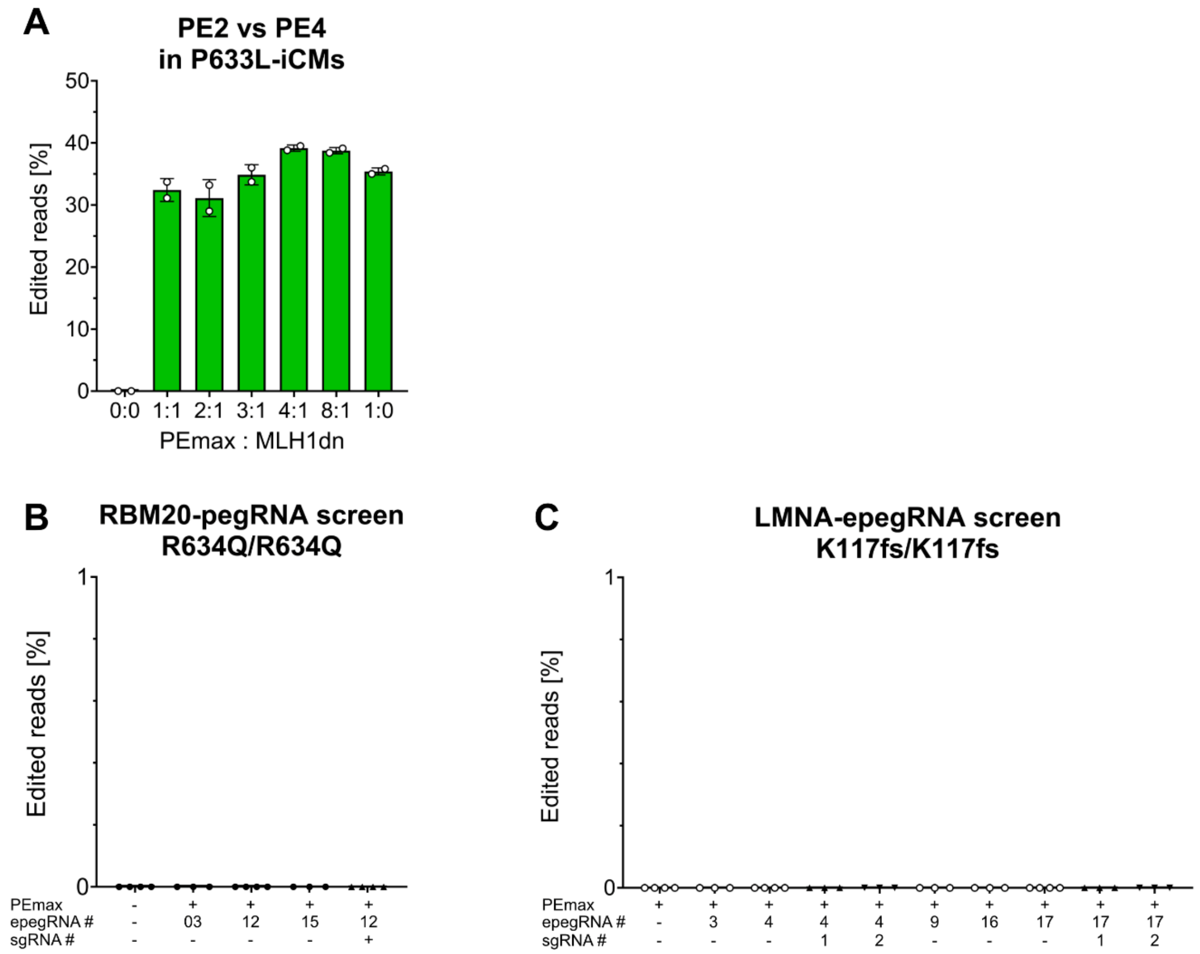

Figure S3: Optimization and epegRNA-screenings in hi-CMs. A Comparison of PE2 versus PE4 editing strategy in homozygous RBM20^{P633L} hi-CMs with epegRNA-12 (n=2 technical replicates). **B** Percentage of A-to-G editing after PE with PEmax and respective epegRNA in hi-CMs carrying the homozygous RBM20^{R634Q}-mutation. Untreated, epegRNA-12, epegRNA-12+sgRNA, n=4 independent experiments. **C** Percentage of edited reads in homozygous LMNA^{K117fs}-hi-CMs after treatment with PE4 or PE5 and indicated epegRNAs. Untreated, epegRNA-04 and -17, n=4 independent experiments. Data are expressed as mean (SD). Three independent experiments (n=3) were performed

unless indicated otherwise. Editing efficiencies were quantified by Sanger sequencing trace deconvolution.

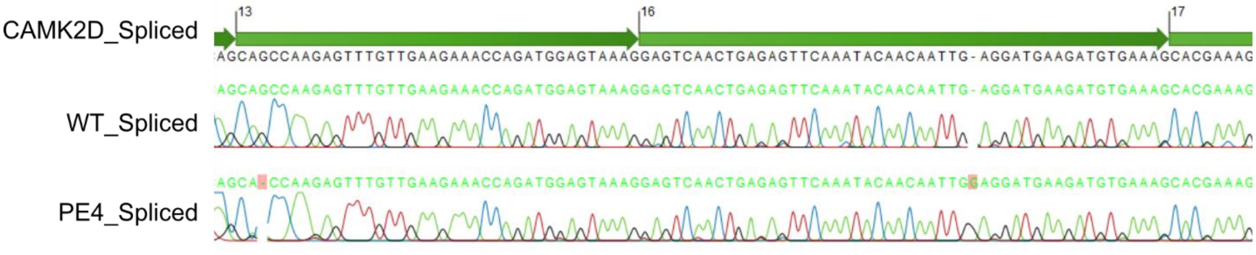

Figure S4: Sequencing validation of the correctly spliced CAMK2D isoform in hi-CMs. Sanger sequencing electropherograms of the wildtype and PE-treated mutant hi-CMs are shown. Exons 13, 16 and 17 are annotated in green.

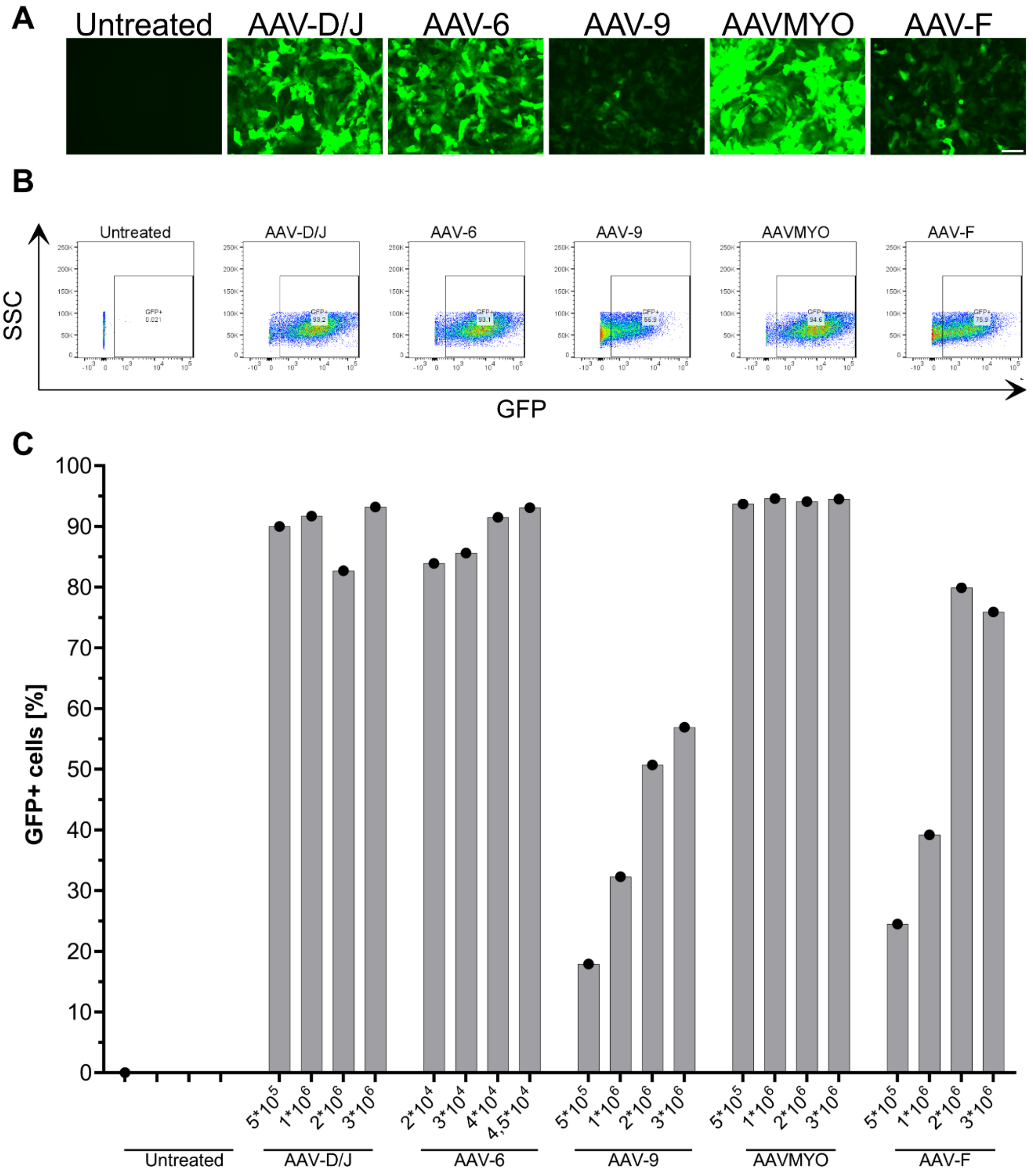

Figure S5: **Quantification of rAAV-transduction efficiencies in hi-CMs with different serotypes. A** Representative fluorescence images of day-21 hi-CMs transduced with GFP-expressing rAAV with respective capsid serotype. Scale bar, 50 µm. **B** Representative flow-cytometry plots showing of GFP+ hi-CMs for each rAAV-serotype. **C**

Quantification of transduction efficiency (% GFP+ cells) by FACS across rAAV-serotypes (n=1 experiment). Level of transduction was quantified as vector genomes per cell (vg/cell).
